# FAM167A is a key molecule to induce BCR-ABL-independent TKI resistance in CML via noncanonical NF-κB signaling activation

**DOI:** 10.1186/s13046-022-02298-1

**Published:** 2022-03-03

**Authors:** Taewoo Yang, Kyu-Young Sim, Gwang-Hoon Ko, Jae-Sook Ahn, Hyeoung-Joon Kim, Sung-Gyoo Park

**Affiliations:** 1 Institute of Pharmaceutical Sciences, College of Pharmacy, Seoul National University, 08826 Seoul, Republic of Korea; 2 School of Life Sciences, Gwangju Institute of Science and Technology, Gwangju, 61005 Republic of Korea; 3 Department of Hematology-Oncology, Chonnam National University Hwasun Hospital, 58128 Hwasun, Republic of Korea

**Keywords:** Chronic myeloid leukemia, FAM167A, Noncanonical NF-κB, Tyrosine kinase inhibitor, Resistance

## Abstract

**Background:**

BCR-ABL-independent drug resistance is a barrier to curative treatment of chronic myeloid leukemia (CML). However, the molecular pathways underlying BCR-ABL-independent tyrosine kinase inhibitor (TKI) resistance remain unclear.

**Methods:**

In silico bioinformatic analysis was performed to identify the most active transcription factor and its inducer that contribute to BCR-ABL-independent TKI resistance. Tandem mass spectrometry analysis was performed to identify the receptor for the noncanonical NF-κB activator FAM167A. In vitro and in vivo mouse experiments revealed detailed molecular insights into the functional role of the FAM167A-desmoglein-1 (DSG1) axis in BCL-ABL-independent TKI resistance. CML cells derived from CML patients were analyzed using quantitative reverse transcription PCR and flow cytometry.

**Results:**

We found that NF-κB had the greatest effect on differential gene expression of BCR-ABL-independent TKI-resistant CML cells. Moreover, we found that the previously uncharacterized protein FAM167A activates the noncanonical NF-κB pathway and induces BCR-ABL-independent TKI resistance. Molecular analyses revealed that FAM167A activates the noncanonical NF-κB pathway by binding to the cell adhesion protein DSG1 to upregulate NF-κB-inducing kinase (NIK) by blocking its ubiquitination. Neutralization of FAM167A in a mouse tumor model reduced noncanonical NF-κB activity and restored sensitivity of cells to TKIs. Furthermore, FAM167A and surface DSG1 levels were highly upregulated in CD34^+^ CML cells from patients with BCR-ABL-independent TKI-resistant disease.

**Conclusions:**

These results reveal that FAM167A acts as an essential factor for BCR-ABL-independent TKI resistance in CML by activating the noncanonical NF-κB pathway. In addition, FAM167A may serve as an important target and biomarker for BCR-ABL-independent TKI resistance.

**Supplementary Information:**

The online version contains supplementary material available at 10.1186/s13046-022-02298-1.

## Background

Chronic myeloid leukemia (CML) is caused by a characteristic t (9;22)(q34;q11) reciprocal translocation of the BCR-ABL Philadelphia chromosome. The *BCR-ABL* fusion gene encodes a BCR-ABL oncoprotein that is constitutively active due to loss of the upstream regulatory domain of ABL1 during the translocation [1, 2]. The development of BCR-ABL-targeting tyrosine kinase inhibitors (TKIs), such as imatinib, has revolutionized the treatment of CML patients [3–5]; however, resistance to TKIs remains a major obstacle to successful CML treatment.

TKI resistance occurs in two different ways: BCR-ABL-dependent and BCR-ABL-independent. BCR-ABL-dependent resistance is caused mainly by mutations in the BCR-ABL kinase domain, although many CML patients without mutations in this domain also show TKI resistance [6–9]. Second-generation TKIs such as nilotinib and dasatinib are effective treatments for BCR-ABL-dependent resistant disease [10]. By contrast, BCR-ABL-independent TKI resistance cannot be addressed by second-generation TKIs [6]. Although several factors, including changes in signaling pathway activity, mutations in epigenetic regulators, microenvironmental factors, and leukemic stem cell activity, are potential contributors to BCR-ABL-independent TKI resistance [11, 12], the reasons underlying this type of resistance are still not fully understood. Therefore, identifying the underlying mechanism(s) and developing effective therapies are major goals.

Nuclear factor-κB (NF-κB) family members are inducible transcription factors that regulate a variety of genes involved in immune responses, inflammation, and cancers [13–18]. There are two major NF-κB activation signaling pathways: canonical and noncanonical. The canonical NF-κB pathway is activated mainly through degradation of inhibitor of κB (IκB) proteins and nuclear translocation of p50-containing dimers [13, 19]. By contrast, the central signaling protein in the noncanonical NF-κB pathway is NF-κB-inducing kinase (NIK) [20, 21]. Noncanonical NF-κB activation relies on accumulation of NIK via blockade of ubiquitination-dependent NIK degradation. Accumulated NIK induces the processing of p100 to p52, which is followed by nuclear translocation of p52-containing dimers [22, 23]. NF-κB pathways contribute to TKI resistance, and inhibition of these pathways restores the sensitivity of CML to TKIs [24, 25]. However, the factors involved in direct activation of the NF-κB pathway in TKI-resistant CML remain unknown.

In this study, we show that NF-κB is the most highly activated transcription factor among those tested via in silico activated transcription factor analysis, and is responsible for differential gene expression in BCR-ABL-independent TKI-resistant CML cells. Furthermore, we reveal the critical function of a previously uncharacterized protein, FAM167A, in the noncanonical NF-κB pathway in CML with BCR-ABL-independent TKI resistance. We show that secreted FAM167A activates the noncanonical NF-κB pathway. Moreover, liquid chromatography tandem mass spectrometry analysis identified the cell adhesion protein desmoglein-1 (DSG1) [26–28] as a receptor for FAM167A. Interestingly, DSG1 inhibits the noncanonical NF-κB pathway, but this inhibition is impaired by dissociation of NIK from the DSG1 complex when FAM167A binds to DSG1. We also show that expression of FAM167A is highly upregulated in CD34^+^ CML cells from patients with BCR-ABL-independent TKI resistance (similar to that in CML cell lines), and that FAM167A is responsible for BCR-ABL-independent TKI resistance: neutralizing FAM167A reversed TKI resistance in cultured cells and in a mouse tumor model. Taken together, these findings describe a novel and crucial role for FAM167A in the noncanonical NF-κB pathway with respect to the mechanism of BCR-ABL-independent TKI resistance, suggesting a promising target for overcoming TKI-resistant CML.

## Methods

### Cell culture

The human CML cell line K562S was obtained from the Korean Cell Line Bank. KCL22S and KCL22R were obtained from the American Type Culture Collection. The K562R cell line was generated from K562S by treating cells with gradually increasing concentrations of TKIs. All cell lines were maintained at 37 °C and 5% CO_2_ in a humidified cell culture incubator and cultured in Roswell Park Memorial Institute (RPMI) 1640 medium (HyClone) supplemented with 10% fetal bovine serum (HyClone), 100 U/ml penicillin, and 100 μg/ml streptomycin. To maintain TKI resistance, K562R and KCL22R cells were cultured in medium supplemented with 1 μM imatinib.

### Primary human samples

Peripheral blood or bone marrow samples were obtained from 58 CML patients (clinicopathological characteristics listed in Additional file 1: Table S1) at Hwasun Chonnam National University Hospital. Samples were obtained at the time of diagnosis and prior to any treatment with TKIs (diagnosis), and again at follow-up after treatment with TKIs (follow-up). Mononuclear cells isolated from the patients were frozen and stored in liquid nitrogen until further use. Informed consent was obtained in accordance with the Declaration of Helsinki, and all procedures were approved by the Institutional Review Board of Seoul National University (E2103/003–007) and the Gwangju Institute of Science and Technology (20180629-BR-36-01-02). TKI-responder and TKI-resistant patients were classified clinically according to the European Leukemia Net guidelines [29]. The mutational status of BCR-ABL in CML samples was confirmed by sequencing analysis.

### Plasmids, antibodies, and reagents

DSG1 and HA-tagged S100A4, CYP11A1, ACSS1, LY6G6D, NR5A1, AIF1L, and FAM167A were cloned into the MigR1 vector. pClneo-Myc-Erbin was obtained from Addgene. pFLAG-NIK-DN was described previously [30]. Anti-FAM167A (sc-393999), anti-glyceraldehyde 3-phosphate dehydrogenase (GAPDH) (sc-166574), anti-p100/p52 (sc-7386 X), anti-DSG1 (sc-59904), and anti-ubiquitin (sc-8017) antibodies were purchased from Santa Cruz Biotechnology; anti-NIK (4994), anti-p100/p52 (4882), anti-Myc (2276), anti-TRAF3 (4729), anti-CHIP (2080), and anti-c-cbl (2747) antibodies were purchased from Cell Signaling Technology; the anti-DSG1 antibody (32–6000) was purchased from Invitrogen; the anti-Erbin antibody (NBP2–13968) was purchased from Novus Biologicals; and the control IgG (H9658) was purchased from Sigma-Aldrich. Imatinib (sc-202180). DSG1-specific short hairpin RNA (shRNA) lentiviral particles (sc-35224-V) and control shRNA lentiviral particles (sc-108080) were obtained from Santa Cruz Biotechnology. Nilotinib (1750) was obtained from BioVision, recombinant FAM167A (MBS1363522) was obtained from MyBioSource, SN50 (481480) and MG132 (474790) were obtained from Sigma-Aldrich, and the Pierce Silver Stain Kit (24612) was obtained from Thermo Fisher Scientific.

### Microarray and RNA-seq analyses

Total RNA was extracted with TRI Reagent (Molecular Research Center). For the microarray, synthesis of cDNA and biotinylated cRNA was performed using the Illumina TotalPrep RNA Amplification Kit (Ambion). Labeled cRNAs were hybridized to the Human HT-12 v4 Expression BeadChip (Illumina), and arrays were scanned with a Bead Array Reader confocal scanner (Illumina). For RNA-seq, libraries were prepared using the TruSeq Stranded mRNA LT Sample Prep Kit (Illumina) in accordance with the Illumina TruSeq protocol. Paired-end sequencing was performed using a NovaSeq 6000 system (Illumina). Genes showing a fold-change ≥2 were defined as differentially expressed and displayed in a heatmap generated by Multiple Experiment Viewer. For in silico analyses of activated transcription factors, information about transcription factors associated with their respective binding sites in differentially expressed or randomly selected gene promoters was obtained from the GeneCards database (https://www.genecards.org/), and plots were generated using Cytoscape. The enrichment score of a transcription factor was calculated using the following equation: enrichment score = (proportion of the transcription factor associated with the differentially expressed genes)/(proportion of the transcription factor associated with randomly selected genes) × (number of differentially expressed genes associated with the transcription factor).

### Luciferase reporter assay

K562S and K562R cells were co-transfected with a Renilla luciferase vector [31] and an NF-κB-dependent reporter construct (pBIIx-luc) [31], or an AP-1-dependent reporter construct (AP-1-luc) [32] and other plasmids, using Lipofectamine 2000 (Invitrogen), and then treated as indicated. Twenty-four hours after transfection, luciferase activity was measured using the Dual-luciferase Reporter Assay Kit (Promega) and normalized to Renilla luciferase activity.

### Quantitative reverse transcription PCR (qRT-PCR)

Total cellular RNA was isolated using the RNeasy Mini Kit (Qiagen) and cDNA was prepared with TOPscript RT Drymix (Enzynomics). qRT-PCR was performed using SYBR Green qPCR 2× Premix (Enzynomics) on a Stratagene Mx3000P (Agilent Technologies). The results were normalized to the level of GAPDH. The qRT-PCR primer sequences were as follows: FAM167A-F, 5′-GCACAGTGAACACAACTAACC-3′; FAM167A-R, 5′-CTTGGGGATGGCAGAGAGAT-3′; S100A4-F, 5′-TCTTGGGGAAAAGGACAGATG-3′; S100A4-R, 5′-CATTTCTTCCTGGGCTGCTTA-3′; CYP11A1-F, 5′-TTCCTGCCAAGACACTGGTG-3′; CYP11A1-R, 5′-GATCCGCCGTCCCAGACA-3′; ACSS1-F, 5′-TTGGAGGTCTGGATCCAGTC-3′; ACSS1-R, 5′-GACAAACTCTCC CTCCCCTA-3′; LY6G6D-F, 5′-TACCTGGAAACCCCCCAGT-3′; LY6G6D-R, 5′-TGCCTGCAGGGGCCACAT-3′; NR5A1-F, 5′-TTCAGCCTGGATTTGAAGTTC-3′; NR5A1-R, 5′-CTTGTGGTACAGGTACTCC-3′; AIF1L-F, 5′-AGGGTCTCAAGAGTTGTCCC-3′; AIF1L-R, 5′-ATACTTGGCAGTCCTCACGTT-3′; DSG1-F, 5′-TGCTGGAGTTGAAAGGCATTA-3′; DSG1-R, 5′-AGTGCAATGTGAAATGGGTCT-3′; Erbin-F, 5′-CAAGTCTCGGTGTTCCCTTT-3′; Erbin-R, 5′-AGATCCATTGTTCCGTGAGG-3′; GAPDH-F, 5′*-*GGAGCGAGATCCCTCCAAAAT-3′; and GAPDH-R, 5′-GGCTGTTGTCATACTTCTCATG-3′.

### Cell fractionation

Cells were suspended in hypotonic buffer (10 mM HEPES, pH 7.9; 10 mM KCl; 1.5 mM MgCl_2_; 0.5 mM DTT; 0.5 mM PMSF) and incubated on ice for 15 min. Then, 0.05% Nonidet P-40 was added and the lysates were passed five times through a 25-gauge needle. After centrifugation (1000×g, 5 min, 4 °C) to sediment the nuclei, the supernatants were collected and separated by high-speed centrifugation (16,000×g, 5 min, 4 °C). The resulting supernatants were stored at − 80 °C as the cytosolic fraction. The pellets containing cell nuclei were washed with hypotonic buffer and suspended in hypertonic buffer (20 mM HEPES, pH 7.9; 420 mM NaCl; 1.5 mM MgCl_2_; 25% glycerol; 0.2 mM EDTA; 0.5 mM DTT; 0.5 mM PMSF; 1 μg/mL leupeptin; 1 μg/mL aprotinin; 1 μg/mL pepstatin A), followed by a 30-min incubation on ice with vortexing every 10 min. After centrifugation of the nuclear lysates (16,000×g, 5 min, 4 °C), the resulting supernatants were stored at − 80 °C as the nuclear fraction.

### Immunoblot analysis

Proteins were separated by sodium dodecyl sulfate (SDS)-polyacrylamide gel electrophoresis on 8–15% gels and then transferred to polyvinylidene difluoride membranes. The membranes were probed with specific antibodies as indicated. Densitometry was performed using ImageJ software with GAPDH as an internal standard.

### Immunoprecipitation

Cells were lysed for 30 min on ice in lysis buffer (20 mM Tris-HCl, pH 8.0; 150 mM NaCl; 1% Triton X-100; 10% glycerol; 2 mM EDTA; 1 μg/ml leupeptin; 1 μg/ml aprotinin; 1 μg/ml pepstatin A; 0.1 mM PMSF). The lysates were then centrifuged for 15 min at 15,000×g and 4 °C and the supernatants were incubated at 4 °C under rotation with specific antibodies. Protein G Sepharose® beads (GE Healthcare) were added and incubated under rotation at 4 °C. After washing four times with lysis buffer, the immunoprecipitates were eluted by boiling in SDS sample buffer and then subjected to immunoblot analysis. Band intensities were analyzed by ImageJ software.

### Electrophoretic mobility shift assay (EMSA)

For the supershift analysis, nuclear fractions were incubated for 20 min at room temperature with an anti-p52 antibody or isotype control in binding buffer (5 mM Tris, pH 7.5; 25 mM KCl; 0.5 mM EDTA; 2.5% glycerol; 0.5 mM DTT; 0.1 μg/μl poly (dI/dC); 0.5 mg/ml BSA), followed by further incubation for 20 min at room temperature with a biotinylated double-stranded NF-κB probe (5′-AGTTGAGGGGACTTTCCCAGG-3′). The reaction samples were separated through 6% non-denaturing polyacrylamide gels and transferred to nylon membranes. The probes on the membranes were visualized using a LightShift Chemiluminescent EMSA kit (Thermo Fisher Scientific) and quantified by ImageJ.

### Flow cytometry analysis and cell sorting

For surface DSG1 staining, cells were either incubated with Myc-tagged FAM167A (MyBioSource), followed by staining with an Alexa Fluor 488-conjugated anti-Myc antibody (9B11, Cell Signaling Technology), or stained with an extracellular domain-targeting anti-DSG1 antibody (129204, R&D Systems), followed by an Alexa Fluor 488-conjugated anti-mouse IgG antibody (Invitrogen). For primary cells, PE-conjugated anti-CD34 (4H11, eBioscience) and anti-DSG1 antibodies (129204, R&D Systems), conjugated to Alexa Fluor 488 using an Alexa Fluor 488 antibody labeling kit, (Invitrogen) were used to stain mononuclear cells. Flow cytometry analysis and cell sorting were performed using a Guava EasyCyte HT (Millipore), a FACSCanto II (BD Biosciences), or FACSAria III (BD Biosciences), and the data were analyzed with FlowJo software (TreeStar).

### Immunofluorescence and immunohistochemical staining

For immunofluorescence staining, fixed and permeabilized cells were stained with an anti-DSG1 antibody (129204, R&D Systems), followed by an Alexa Fluor 488-conjugated anti-mouse IgG antibody (Invitrogen). Nuclei were visualized by Hoechst 33342 (Sigma-Aldrich) staining. An FV1000 confocal microscope (Olympus) and accompanying FV10-ASW software were used to acquire confocal images. For immunohistochemical staining, fixed and paraffin-embedded tumor sections were stained with hematoxylin and eosin (HE), anti-Ki-67 antibody (SP6, Abcam), and anti-NIK antibody (Abcam), using standard procedures. TUNEL staining was performed with the DeadEnd Colorimetric TUNEL System (Promega). Images were captured with an Eclipse Ti inverted microscope (Nikon) and accompanying NIS-Elements software. Quantification was performed using ImageJ software. The number of Ki-67^+^ or TUNEL^+^ cells in each field was counted. NIK expression in tumor cells was scored (intensity: 0 = negative, 1 = weak, 2 = moderate, 3 = strong), and the H score was calculated using the following equation: H score = 1 × (% of cells with intensity 1) + 2 × (% of cells with intensity 2) + 3 × (% of cells with intensity 3).

### Cell viability and apoptosis assays

For the cell viability assays, K562S or K562R cells were seeded in 96-well plates and treated as indicated prior to transient transfection with expression constructs. After 72 h, cell viability was measured using WST reagent (DoGenBio). For the apoptosis assays, K562S or K562R cells were seeded in 6-well plates and treated as indicated. After 72 h, cells were stained with Annexin V-APC and propidium iodide using an Annexin V-APC apoptosis detection kit (Thermo Fisher Scientific) and analyzed on a Guava EasyCyte HT cytometer (Millipore).

### Mouse xenograft model

K562R cells (1× 10^7^) were suspended in 50% (v/v) serum-free Matrigel (Corning) and implanted subcutaneously into the right flanks of 6-week-old female BALB/c (nu/nu) mice. Tumor size was measured with calipers, and the tumor volume was calculated using a standard formula (width^2^ × length/2). When the tumor volume reached 100–200 mm^3^, the mice were randomly divided into groups and treated for 10 days with imatinib (10 mg/kg/day, intraperitoneally), an anti-FAM167A antibody or isotype control (2 mg/kg/3 days, intraperitoneally), or vehicle (saline, PBS). All animal experiments were performed according to protocols approved by Institutional Animal Care and Use Committees at Seoul National University (SNU-210315-6) and the Gwangju Institute of Science and Technology (GIST-2019-008).

### Statistical analysis

The number of replicates for each experiment are indicated in the figure legends. Data are presented as the mean ± standard deviation (s.d.). Two-tailed unpaired Student’s *t*-tests were used to determine statistical significance. *P* values < 0.05 were considered significant (*P < 0.05, **P < 0.01, and ***P < 0.001).

## Results

### NF-κB is the transcription factor predominantly responsible for differential gene expression in BCR-ABL-independent TKI-resistant cells

**Fig. 1:**
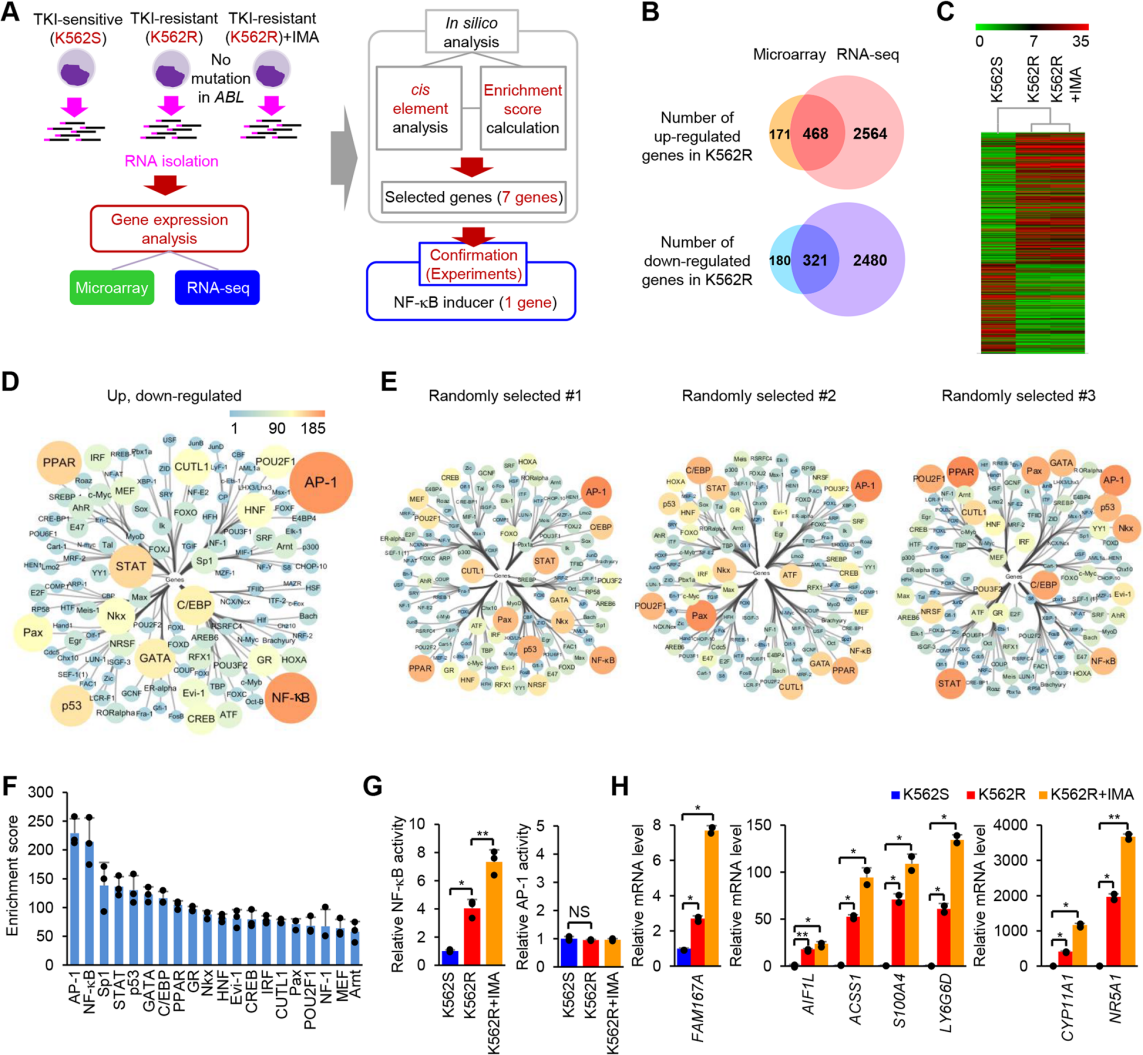
NF-κB is responsible for gene expression changes in K562R cells. **A** Strategy for selection of genes contributing BCR-ABL-independent TKI resistance in CML. **B** Venn diagram showing upregulated and downregulated genes (fold-change ≥2) in K562R cells as examined by microarray and RNA-seq analyses. **C** Heat map analysis of expression levels of genes differentially expressed in K562R cells. **D**, **E** In silico analysis of transcription factors involved in regulating differentially expressed genes (in both the microarray and RNA-seq analyses) (**D**) or of three control group genes (each group contains 500 randomly selected genes) (**E**). The size and color represent the number of genes associated with the transcription factor. **F** Enrichment scores for transcription factors represent the degree of association with the differentially expressed genes compared to randomly selected genes, as detailed by the calculation listed in the Methods. **G** NF-κB and AP-1 luciferase reporter activities in K562S and K562R cells and in K562R cells treated for 24 h with 1 μM imatinib. **H** Quantitative reverse transcription PCR (qRT-PCR) analysis of the mRNA levels of 7 genes in K562S and K562R cells and in K562R cells treated with 1 μM imatinib for 24 h. IMA, imatinib. Data are representative of three (**G**, **H**) independent experiments (error bars, s.d. of duplicate (**H**) or triplicate (**F**, **G**) samples). Unpaired two-tailed *t*-test; *P < 0.05, **P < 0.01. NS, not significant; K562S, TKI-sensitive K562 cell line; K562R, BCR-ABL kinase domain mutation-free TKI-resistant K562 cell line

TKI resistance that does not involve mutations in the BCR-ABL kinase domain (i.e., BCR-ABL-independent TKI resistance) remains poorly understood [6–9]. Initially, we sought to find genes with potential roles in BCR-ABL-independent TKI resistance by identifying those that are differentially expressed in TKI-resistant CML cells (Fig. 1**A**). Therefore, we generated K562R, a TKI-resistant CML cell line without mutations in the BCR-ABL kinase domain (Additional file 1: Fig. S1**A**-**D** and Additional file 1: Fig. S2) and then analyzed gene expression in TKI-sensitive K562S cells, K562R cells, and K562R cells treated with imatinib; both microarray and RNA-sequencing (RNA-seq) analyses were conducted, to eliminate false positives due to analytical methods. Thus, 789 differentially expressed (i.e., fold-change ≥2) genes between K562S and K562R were rigorously selected (Fig. 1**B**, **C**). In addition to identifying differentially expressed genes, we sought to uncover transcription factors that might regulate the differentially expressed genes specifically in BCR-ABL-independent resistant CML cells. In silico analysis of transcription factors responsible for differential gene expression of BCR-ABL-independent TKI-resistant CML cells revealed that AP-1 and NF-κB were the most enriched transcription factors among regulators of differentially expressed genes compared with randomly selected genes (Fig. 1D-F and Additional file 1: Fig. S3). AP-1 and NF-κB showed remarkably higher enrichment scores compared to other transcription factors, and only scores for those two transcription factors were beyond three standard deviations from the mean. To confirm differences in the activity of AP-1 and NF-κB in the TKI-resistant cell line, we performed AP-1 and NF-κB reporter assays in K562R and K562S cells, as well as in imatinib-treated K562R cells, to investigate the effects of TKI treatment. The results showed that NF-κB activity, but that of not AP-1, was significantly higher in K562R cells than in K562S cells; in addition, this activity was much higher in K562R cells treated with imatinib, while there was no significant difference in AP-1 activity (Fig. 1G); this suggests that NF-κB contributes to BCR-ABL-independent TKI resistance by regulating gene expression. In addition, to identify genes that are related to increased NF-κB activity in K562R cells, we selected seven differentially expressed genes (*FAM167A*, *AIF1L*, *ACSS1*, *S100A4*, *LY6G6D*, *CYP11A1*, *NR5A1*) that showed expression profiles similar to NF-κB activity patterns using gene expression profile data from K562S, K562R, and K562R cells treated with imatinib. The expression levels of the selected genes were verified by qRT-PCR (Fig. 1H).

### FAM167A activates the noncanonical NF-κB pathway in BCR-ABL-independent TKI-resistant cells

**Fig. 2:**
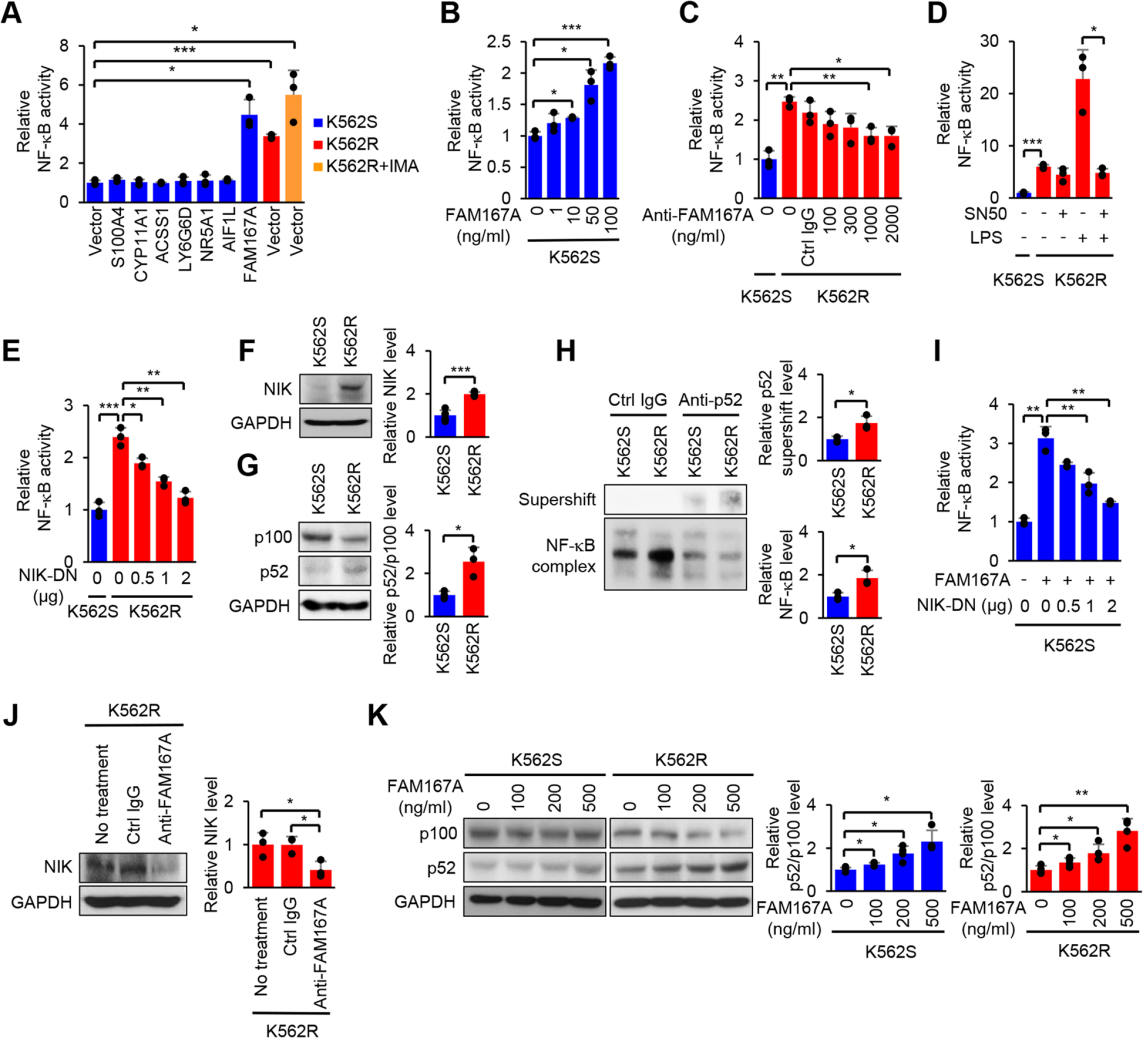
FAM167A is an inducer of the noncanonical NF-κB pathway. **A** NF-κB luciferase reporter activity in K562S cells 24 h after transfection of the plasmid encoding the indicated gene, and in K562R cells and K562R cells treated with 1 μM imatinib for 24 h. **B** NF-κB luciferase reporter activity for 24 h in K562S cells after treatment with recombinant FAM167A. **C** NF-κB luciferase reporter activity in K562R cells after treatment for 24 h with anti-FAM167A neutralizing antibody. **D** NF-κB luciferase reporter activity in K562R cells after treatment for 24 h with 50 μg/ml SN50, 10 ng/ml LPS, or both. **E** NF-κB luciferase reporter activity in K562R cells 24 h after transfection of the plasmid encoding dominant-negative NIK (NIK-DN). **F**, **G** Immunoblot analyses for NIK (**F**) and p100/p52 (**G**) in K562S and K562R cells. **H** Electrophoretic mobility shift assay (EMSA) of nuclear extracts isolated from K562S and K562R cells showing super shift with an NF-κB probe and an anti-p52 antibody. **I** NF-κB luciferase reporter activity in K562S cells 24 h after treatment with 100 ng/ml recombinant FAM167A and transfection of the plasmid encoding NIK-DN. **J** Immunoblot analysis of NIK in K562R cells after treatment with 2 μg/ml of anti-FAM167A neutralizing antibody or an isotype control for 12 h. **K** Immunoblot analysis of p100/p52 in K562S and K562R cells after treatment for 12 h with the indicated concentrations of recombinant FAM167A. GAPDH was used as an internal standard (**F**, **G**, **J**, **K**). Data are representative of three (**A**-**E**, **G**-**K**) or five (**F**) independent experiments (error bars, s.d. of triplicate (**A**-**E**, **G**-**K**) or five (**F**) samples). Unpaired two-tailed *t*-test; *P < 0.05, **P < 0.01, ***P < 0.001

To screen selected genes, we measured NF-κB activity in K562S cells transfected with a plasmid encoding the indicated gene. Among the seven selected genes, only FAM167A increased NF-κB activity significantly when expressed ectopically (Fig. 2A). In addition, compared to other genes, ectopic expression of FAM167A increased the viability of K562S cells in the presence of imatinib (Additional file 1: Fig. S4). Even though FAM167A does not have signal sequences for secretion, bioinformatic analysis with SecretomeP (http://www.cbs.dtu.dk/services/SecretomeP/), which predicts nonclassical protein secretion, showed that FAM167A has a high score (NN-score = 0.905) (Additional file 1: Fig. S5**A**). Thus, we analyzed secretion of FAM167A by immunoblot analyses of K562R cell lysates and culture supernatants, and confirmed that FAM167A is secreted by these cells (Additional file 1: Fig. S5**B**). To test the NF-κB activating function directly, we used a recombinant FAM167A protein and a neutralizing antibody specific for FAM167A to increase and reduce, respectively, FAM167A levels during cell culture. Consistent with the bioinformatic analysis results, treatment of K562S cells with soluble recombinant FAM167A increased NF-κB activity (Fig. 2B), while neutralizing FAM167A in K562R cell cultures reduced NF-κB activity (Fig. 2C), suggesting that the secreted FAM167A protein functions as an inducer of the NF-κB pathway.

Next, we aimed to determine which NF-κB pathway is activated in K562R cells; to do this, cells were treated with inhibitors of specific NF-κB pathways. Treatment with SN50, which inhibits the canonical NF-κB pathway, did not reduce NF-κB activity in K562R cells significantly, even though SN50 treatment blocked a further lipopolysaccharide (LPS)-stimulated increase in NF-κB activity (Fig. 2D). By contrast, transient expression of dominant-negative NIK (NIK-DN), which inhibits the noncanonical NF-κB pathway, reduced NF-κB activity in K562R cells significantly and to a level similar to that observed in unstimulated K562S cells (Fig. 2E). Consistent with these results, the level and activity of NIK for processing p100 into p52 were higher in K562R cells than in K562S cells (Fig. 2F, G). A supershift analysis performed in an EMSA also showed higher NF-κB activity and p52 supershift levels in K562R cells (Fig. 2H and Additional file 1: Fig. S6). Based on these findings, we asked whether FAM167A is responsible for activation of the noncanonical NF-κB pathway. Interestingly, NF-κB activity stimulated by recombinant FAM167A was reduced in cells with transient expression of NIK-DN (Fig. 2I). Consistent with this, neutralization of FAM167A reduced the NIK level in K562R cells significantly (Fig. 2J), and treatment of K562S and K562R cells with recombinant FAM167A increased processing of p100 to p52 (Fig. 2K). Thus, the data indicate that FAM167A activates the noncanonical NF-κB pathway in K562R cells to a greater extent than in K562S cells.

### DSG1 is a FAM167A receptor with relatively high surface expression in BCR-ABL-independent TKI-resistant cells

**Fig. 3:**
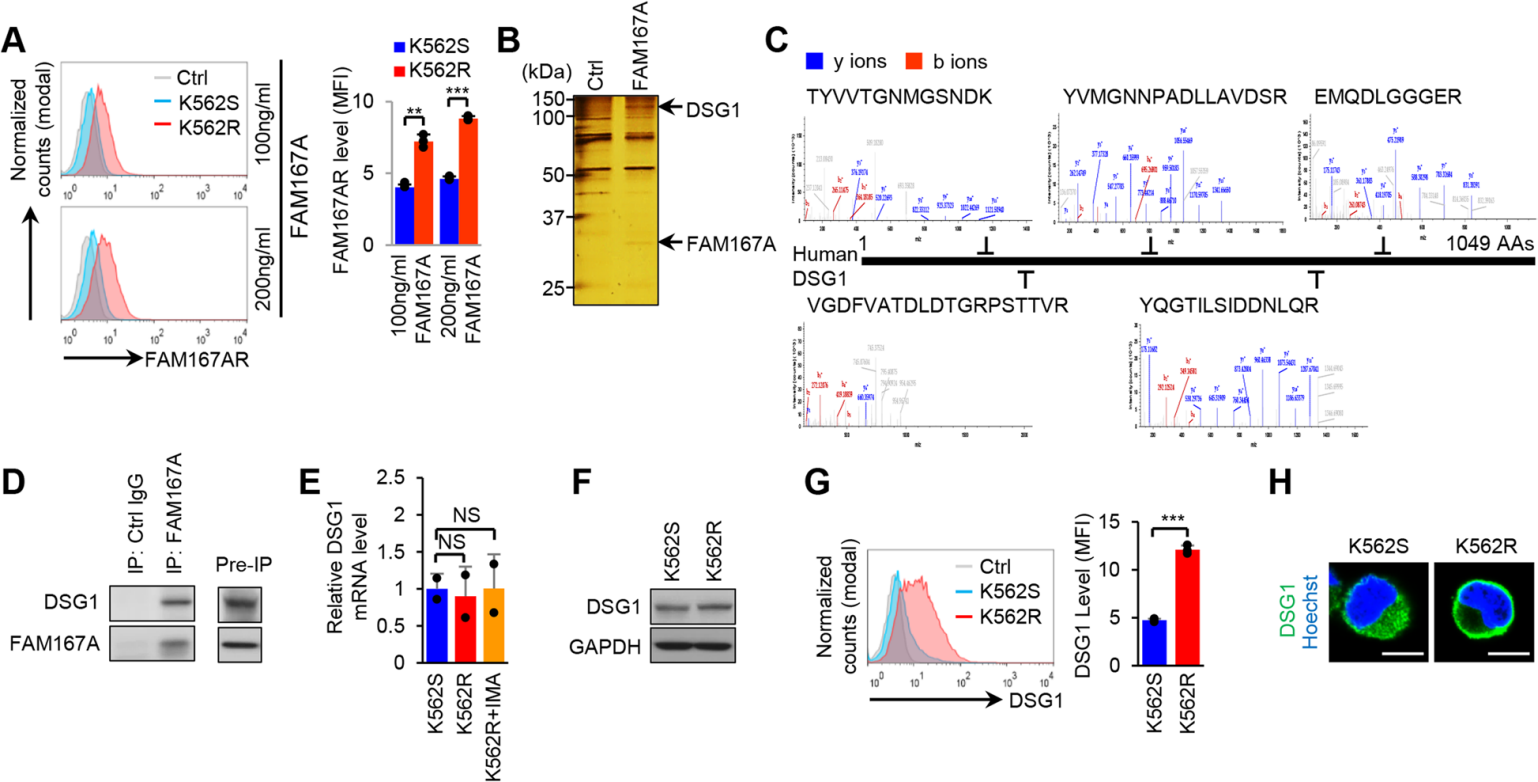
FAM167A binds to its receptor DSG1. **A** Surface FAM167A receptor (FAM167AR) staining of K562S and K562R cells using Myc-tagged FAM167A and an Alexa Fluor 488-conjugated anti-Myc antibody. **B** Silver staining of immunoprecipitated samples prepared from K562R cells using Myc-tagged FAM167A and an anti-Myc antibody bound to Protein G Sepharose® beads. **C** MS/MS spectrum and peptide sequence of the indicated band from **B**. **D** Co-immunoprecipitation analysis between FAM167A and DSG1 in K562R cells. **E** qRT-PCR analysis of DSG1 mRNA levels in K562S and K562R cells, and in K562R cells treated with 1 μM imatinib for 24 h. **F** Immunoblot analysis of DSG1 expression in K562S and K562R cells. **G** Surface staining of K562S and K562R cells using an anti-DSG1 antibody. **H** Immunofluorescence microscopy analysis of DSG1 expression in K562S and K562R cells. Nuclei were visualized using Hoechst 33342. Scale bars, 10 μm. GAPDH was used as an internal standard (**F**). IP, immunoprecipitation. MFI, mean fluorescence intensity. Data are representative of three (**A**, **B**, **D**-**H**) independent experiments (error bars, s.d. of duplicate (**E**) or triplicate (**A**, **G**) samples). Unpaired two-tailed *t*-test; **P < 0.01, ***P < 0.001. NS, not significant

As treatment with and neutralization of secreted FAM167A effectively regulated the noncanonical NF-κB pathway, we hypothesized that a receptor for FAM167A might be expressed on the cell surface. To evaluate expression of potential receptors for FAM167A, we first examined FAM167A levels at the cell surface by labeling K562S and K562R cells with Myc-tagged recombinant FAM167A, followed by staining with a fluorescent dye-conjugated anti-Myc antibody and flow cytometry. The results suggested that a receptor for FAM167A was expressed on the cell surface, and that levels on K562R cells were higher than those on K562S cells (Fig. 3A). After verification of the presence of the receptor on the cell surface and prior to isolation of the receptor for identification, we pulled down the receptor from K562R cells using Myc-tagged recombinant FAM167A and anti-Myc antibodies bound to Protein G Sepharose® beads (schematic shown in Additional file 1: Fig. S7**A**). Silver staining of proteins immunoprecipitated with Myc-tagged FAM167A showed two distinct bands compared with background that was also detected in control samples immunoprecipitated without the FAM167A (Fig. 3B and Additional file 1: Fig. S7**B**). LC-MS/MS analysis identified one band (around 30 kDa) as recombinant FAM167A and the other band (around 120 kDa) as DSG1, a known cell adhesion molecule [26–28] (Fig. 3C and Additional file 1: Fig. S7**C**). Co-immunoprecipitation experiments with K562R cell lysates confirmed that FAM167A binds to DSG1 (Fig. 3D). To verify that upregulated surface expression of DSG1 was mediated by increases in total DSG1 protein levels, we measured expression of DSG1 mRNA and protein. Interestingly, there were no differences in expression of either mRNA or protein between K562S and K562R cells (Fig. 3E, F). By contrast, cell surface expression of DSG1 was higher in K562R cells (Fig. 3A, G). Immunofluorescence staining confirmed that DSG1 was mainly localized on the surface of K562R cells, but not K562S cells (Fig. 3H). Furthermore, FAM167A was mainly colocalized with DSG1 on the surface of K562R cells (Additional file 1: Fig. S7**D**). Although FAM167A was ectopically expressed in K562S cells, FAM167A-induced NF-κB activation was significantly inhibited by treatment with anti-FAM167A neutralizing antibody (Additional file 1: Fig. S7**E**), indicating that secreted FAM167A activates the noncanonical NF-κB pathway. Taken together, these results show that DSG1 is a receptor of FAM167A. and that its surface expression is higher in K562R cells than in K562S cells.

### FAM167A regulates NIK ubiquitination through DSG1

**Fig. 4:**
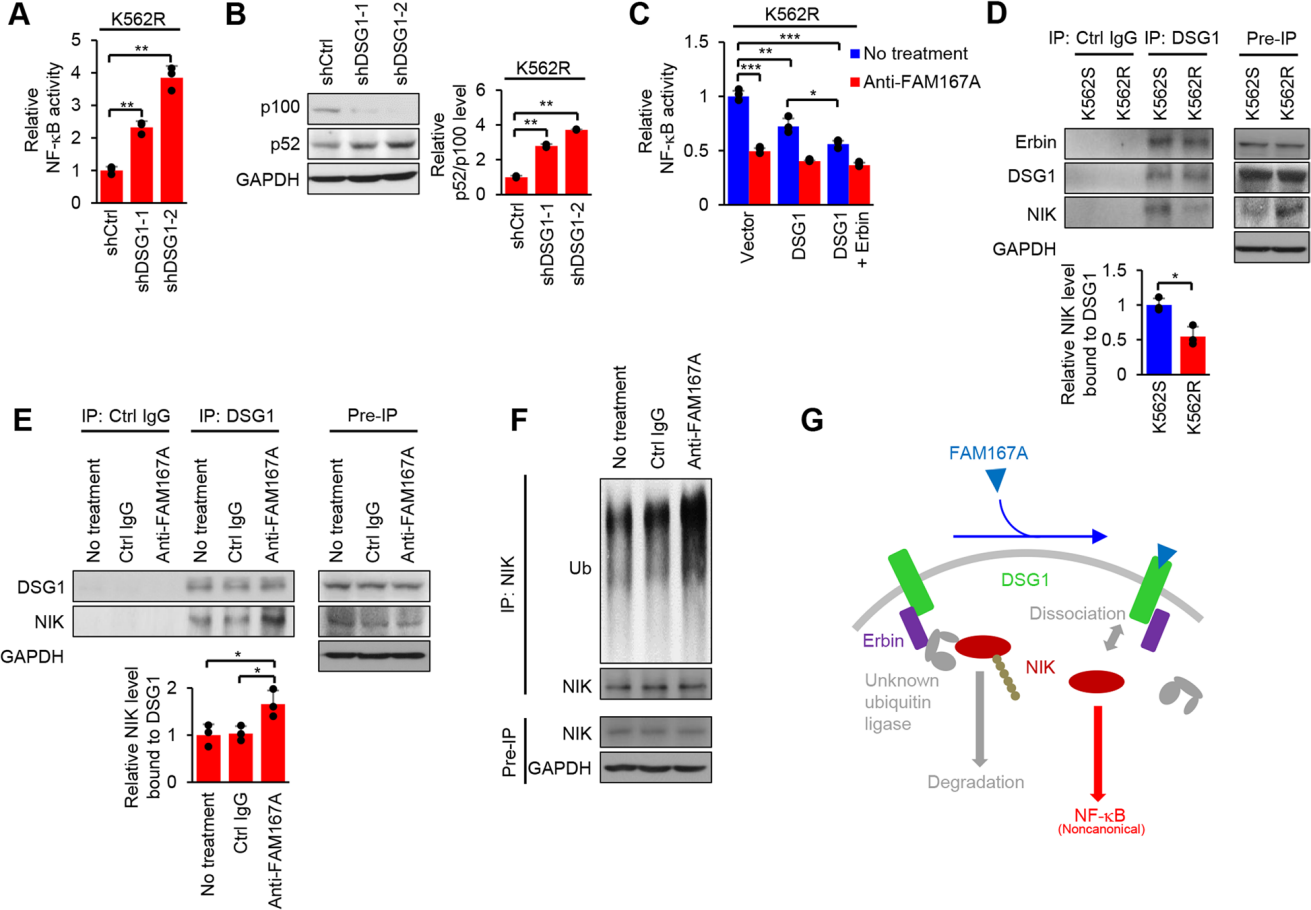
FAM167A regulates NIK binding and ubiquitination through DSG1. **A** NF-κB luciferase reporter activity in K562R cells after DSG1 knockdown using recombinant lentiviruses encoding two different DSG1-specific shRNAs. **B** Immunoblot analysis of p100/p52 in K562R cells after DSG1 knockdown. **C** NF-κB luciferase reporter activity in K562R cells 24 h after transfection of a plasmid encoding DSG1, or both DSG1 and Erbin, with or without treatment with 2 μg/ml anti-FAM167A neutralizing antibody. **D** Co-immunoprecipitation analysis of DSG1, Erbin, and NIK in K562S and K562R cells. **E** Co-immunoprecipitation analysis of DSG1 and NIK in K562R cells after treatment with 2 μg/ml of an anti-FAM167A neutralizing antibody or an isotype control for 3 h. **F** Immunoblot analysis of NIK ubiquitination with an anti-ubiquitin antibody after immunoprecipitation of NIK from K562R cells treated with 2 μg/ml of an anti-FAM167A neutralizing antibody or an isotype control plus 10 μM MG132 for 3 h. **G** Schematic model of the FAM167A-induced noncanonical NF-κB pathway. GAPDH was used as an internal standard (**B**, **D**-**F**). Ub, ubiquitination. Data are representative of two (**B**) or three (**A**, **C**-**F**) independent experiments (error bars, s.d. of duplicate (**B**) or triplicate (**A**, **C**-**E**) samples). Unpaired two-tailed *t*-test; *P < 0.05, **P < 0.01, ***P < 0.001

Because surface expression of DSG1 was higher in K562R cells than in K562S cells (Fig. 3**A**, **G**), we hypothesized that DSG1 knockdown in K562R cells might reduce noncanonical NF-κB activity. To test this, K562R cells were lentivirally transduced with DSG1-specific shRNA constructs to knock down expression of DSG1 (Additional file 1: Fig. S8**A**). Contrary to our expectations, DSG1 knockdown increased NF-κB activity and processing of p100 into p52 in K562R cells (Fig. 4A, B). To confirm these results, we measured NF-κB activity after transient expression of DSG1 and its interacting protein Erbin [33] in K562R cells. NF-κB activity was reduced by transient expression of DSG1, and further reduced by transient expression of both DSG1 and Erbin (Fig. 4C). Moreover, transient expression of DSG1 and Erbin in the presence of FAM167A-neutralizing antibodies caused very little further reduction in NF-κB activity (not significant, *p* > 0.05) (Fig. 4C). These data demonstrate that DSG1 and Erbin inhibit, whereas FAM167A activates, noncanonical NF-κB via the same pathway.

To further investigate the molecular mechanism underlying this pathway, we first examined the expression levels of Erbin in K562S and K562R cells. The mRNA and protein levels of Erbin were not significantly different between K562S and K562R cells (Additional file 1: Fig. S8**B**, **C**), similar to the DSG1 mRNA and protein levels (Fig. 3E, F). Therefore, we next examined the interactions among DSG1, Erbin, and NIK, as NIK is a key component of the noncanonical NF-κB pathway [34]. Co-immunoprecipitation analyses showed that binding of Erbin to DSG1 was not significantly different between K562S and K562R cells, but that binding of NIK to DSG1 was reduced in K562R cells (Fig. 4D). To further determine whether FAM167A causes a change in NIK binding to DSG1, we neutralized FAM167A protein in K562R cells and assessed the binding of NIK to DSG1; we observed increased binding when FAM167A was neutralized (Fig. 4E).

Moreover, because NIK is regulated via ubiquitination-mediated degradation [22], we were interested in examining NIK ubiquitination after FAM167A neutralization in K562R cells. Immunoblot analysis showed that ubiquitination of NIK increased when FAM167A was neutralized (Fig. 4F). Although ubiquitination of NIK appeared to be regulated by changes in its interactions with DSG1 and Erbin (Fig. 4D-F), these proteins have no reported ubiquitin ligase functions. To identify the ligase involved in NIK ubiquitination, we performed co-immunoprecipitation assays for candidate ubiquitin ligase components, including TRAF3 [35], CHIP [36], and c-cbl [37]; however, we found no interactions (Additional file 1: Fig. S9**A-D**). Overall, these results suggest that FAM167A activates the noncanonical NF-κB pathway by regulating the binding of NIK to DSG1 and Erbin, as well as its subsequent ubiquitination, which may explain the relationship between noncanonical NF-κB activity and cell surface expression of DSG1 (Fig. 4G).

### FAM167A is responsible for BCR-ABL-independent TKI resistance

**Fig. 5:**
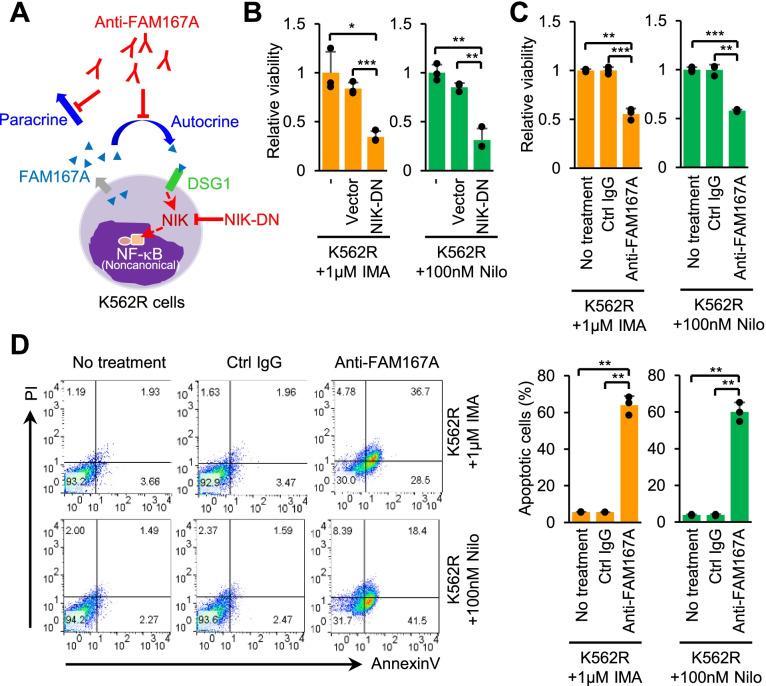
FAM167A contributes to BCR-ABL-independent TKI resistance in vitro. **A** Schematic diagram depicting inhibition of the FAM167A-induced noncanonical NF-κB pathway. **B** Viability of K562R cells transfected with a plasmid encoding NIK-DN after treatment with imatinib or nilotinib (Nilo) for 3 days. **C** Viability analysis of K562R cells after treatment with 2 μg/ml of anti-FAM167A neutralizing antibody or an isotype control for 3 days in the presence of imatinib or nilotinib. **D** The apoptotic population of K562R cells after treatment with 2 μg/ml of anti-FAM167A neutralizing antibody or an isotype control for 3 days in the presence of imatinib or nilotinib. Data are representative of three (**B**-**D**) independent experiments (error bars, s.d. of triplicate (**B**-**D**) samples). Unpaired two-tailed *t*-test; *P < 0.05, **P < 0.01, ***P < 0.001

To investigate whether FAM167A is responsible for BCR-ABL-independent TKI resistance, we first examined the role of the noncanonical NF-κB pathway. Inhibiting the noncanonical NF-κB pathway by transient expression of NIK-DN effectively reduced the viability of K562R cells in the presence of TKIs (Fig. 5A, B). Consistent with this, analysis of cell viability (in a WST assay) and apoptosis (measured by flow cytometry) showed that neutralization of FAM167A reduced the viability of K562R cells significantly (Fig. 5A, C) and increased apoptosis in the presence of TKIs (Fig. 5D). Thus, FAM167A-mediated activation of the noncanonical NF-κB pathway is crucial for TKI resistance.

**Fig. 6:**
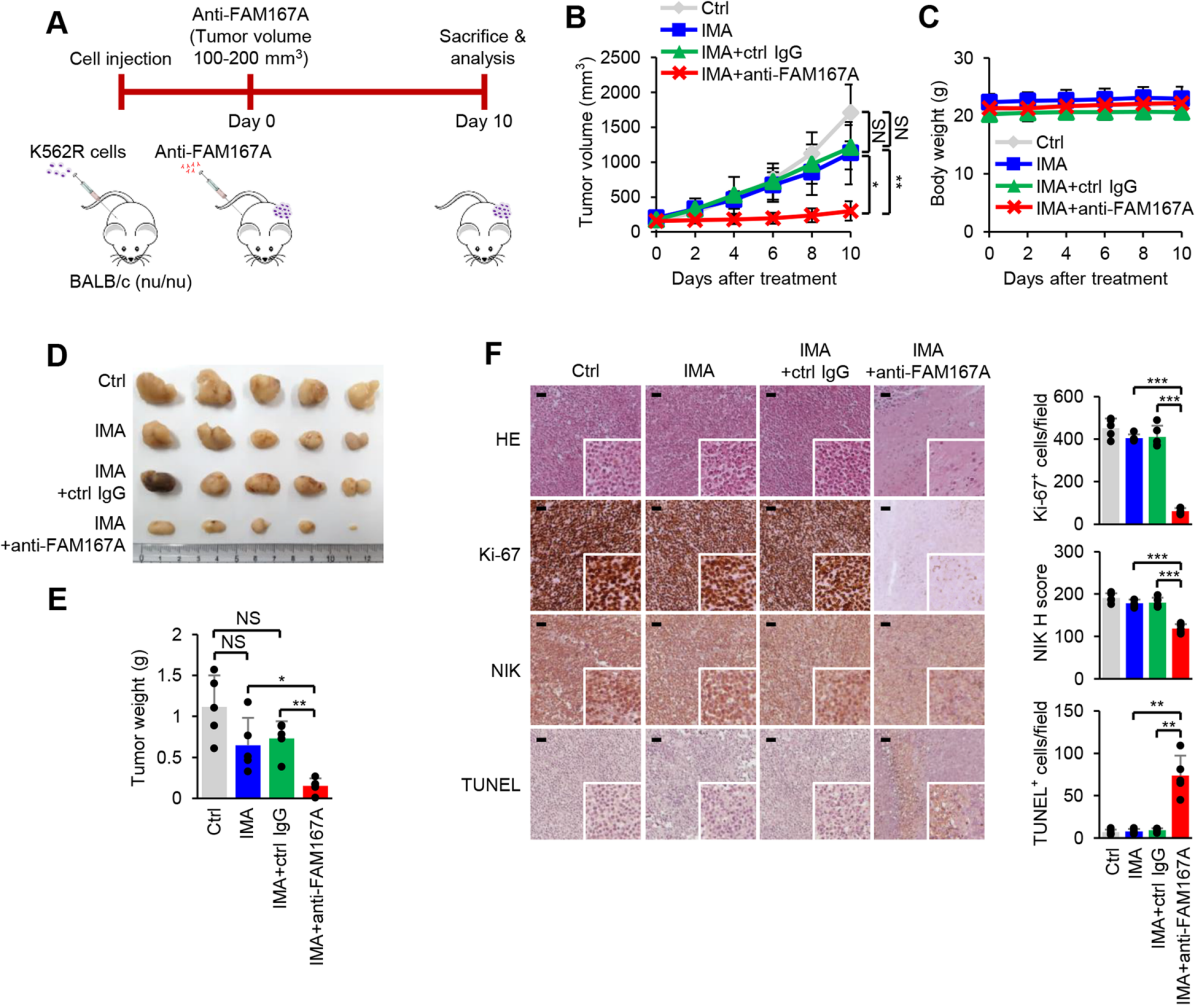
FAM167A contributes to BCR-ABL-independent TKI resistance in vivo. **A** Schedule for experiments and schematic showing the mouse model. **B** Volume of established K562R tumors in mice treated with vehicle or 10 mg/kg/day imatinib with or without the anti-FAM167A neutralizing antibody or isotype control (2 mg/kg/3 days). **C** Body weight of the mice. **D** Photograph of tumors collected from the mice in each group on Day 10 of treatment. **E** Tumor weight on Day 10 of treatment. **F** Hematoxylin and eosin (HE) staining, immunohistochemical staining of tumor sections with anti-Ki-67 and anti-NIK antibodies, and TUNEL staining. Scale bars, 100 μm. Data are representative of three (**B**-**F**) independent experiments (error bars, s.d. of five (**B**, **C**, **E**, **F**) mice or samples). Unpaired two-tailed *t*-test; *P < 0.05, **P < 0.01, ***P < 0.001. NS, not significant

Additionally, we examined the effects of FAM167A on TKI resistance in vivo using a mouse xenograft model established by subcutaneous injection of K562R cells into nude mice (Fig. 6A). Compared with imatinib alone, or imatinib plus an isotype control antibody, treatment with imatinib plus FAM167A neutralization (induced by intraperitoneal injection of an anti-FAM167A neutralizing antibody) led to marked suppression of tumor growth with no loss of body weight (Fig. 6B-E). To investigate the basis of differences in tumor growth at the cellular level, we analyzed tumor tissue sections via HE staining, the results of which showed a lower cell density in FAM167A-neutralized tumors. Further immunohistochemical staining showed reduced expression of NIK and the proliferation marker Ki-67, alongside increased levels of the apoptotic marker TUNEL (Fig. 6F). Taken together, these data suggest that neutralizing FAM167A restores TKI sensitivity in cells with BCR-ABL-independent TKI resistance.

### FAM167A and surface DSG1 levels are elevated in patients with BCR-ABL-independent resistant CML

**Fig. 7:**
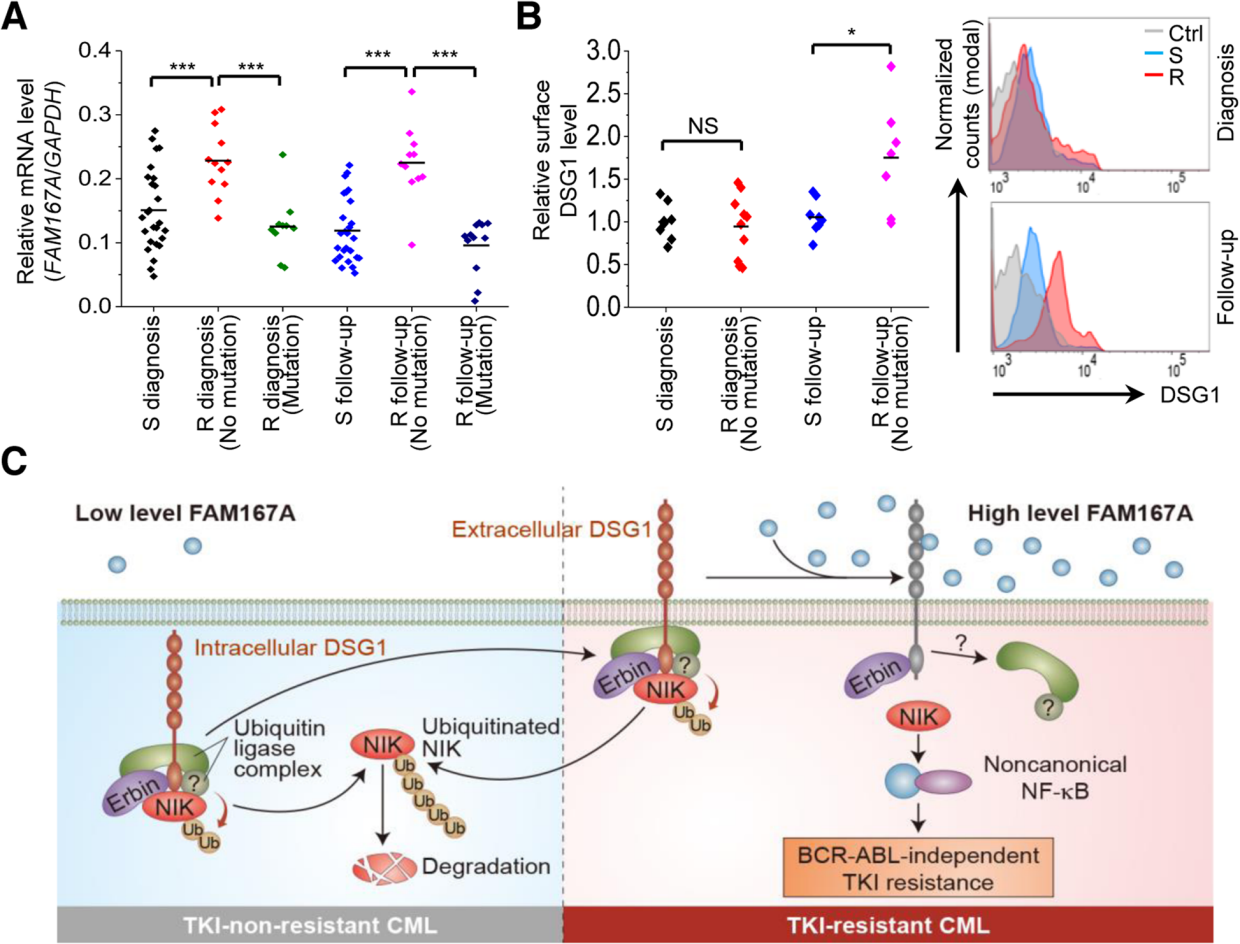
FAM167A and surface DSG1 levels are associated with BCR-ABL-independent TKI resistance in CML patients. **A** qRT-PCR analysis of FAM167A mRNA expression in CD34^+^ stem/progenitor cells from imatinib-responsive CML patients (S) at the time of diagnosis (*n* = 26) and at follow-up (*n* = 26), in imatinib-resistant CML patients (R) without BCR-ABL mutation at the time of diagnosis (*n* = 12) and at follow-up (*n* = 11), and in imatinib-resistant CML patients harboring a BCR-ABL mutation at the time of diagnosis (*n* = 10) and at follow-up (*n* = 12). Imatinib resistance and BCR-ABL mutation status were determined at follow-up. The level of FAM167A was normalized to that of GAPDH. **B** Relative surface expression of DSG1 (MFI) in CD34^+^ stem/progenitor cells from imatinib-responsive CML patients at the time of diagnosis (*n* = 8) and at follow-up (*n* = 8), and from imatinib-resistant CML patients without BCR-ABL mutation at the time of diagnosis (*n* = 10) and at follow-up (*n* = 7), as measured via flow cytometry. **C** Proposed model showing involvement of the FAM167A-induced noncanonical NF-κB pathway in BCR-ABL-independent TKI resistance. Unpaired two-tailed *t*-test; *P < 0.05, ***P < 0.001. NS, not significant

In addition to K562R cells, we examined BCR-ABL-independent TKI-resistant KCL22R cells. Sequence analysis confirmed the absence of mutations in the ABL region (Additional file 1: Fig. S10**A**-**C**). Furthermore, KCL22R cells showed a higher FAM167A level than KCL22S cells, and imatinib treatment of KCL22R cells showed increased FAM167A expression (Additional file 1: Fig. S10**D**). Finally, to determine the clinical importance of our findings, we analyzed FAM167A levels in CD34^+^ stem/progenitor cells isolated from CML patients (Additional file 1: Table S1) by qRT-PCR. The data showed that the expression of FAM167A was higher in cells from BCR-ABL-independent imatinib-resistant patients (R, no mutation) than in cells from either imatinib-responder or BCR-ABL-dependent imatinib-resistant patients (S and R mutations, respectively) (Fig. 7A). In addition, FAM167A levels were higher in cells from BCR-ABL-independent imatinib-resistant patients before they received treatment (i.e., at diagnosis), suggesting that expression of FAM167A is predictive of BCR-ABL-independent TKI resistance in CML patients. Flow cytometry analysis also showed that surface expression of DSG1 was higher in cells from BCR-ABL-independent imatinib-resistant patients after imatinib treatment (follow-up) than before treatment (diagnosis) (Fig. 7B). Collectively, these findings suggest that FAM167A and DSG1 are associated with BCR-ABL-independent TKI resistance in CML patients via activation of the noncanonical NF-κB pathway (Fig. 7C).

## Discussion

Initially, TKIs were thought to be a successful form of targeted cancer therapy [3–5]. However, later studies showed that TKIs are not completely curative because cells develop BCR-ABL-independent TKI resistance; therefore, an additional therapeutic strategy is required [6]. Our study identifies a previously uncharacterized protein, FAM167A, that is upregulated in cells with BCR-ABL-independent TKI resistance. This protein induces resistance by activating the noncanonical NF-κB pathway. In addition, our analyses of clinical samples showed that FAM167A is upregulated in cells from BCR-ABL-independent resistant patients, not only after TKI treatment but also at the time of diagnosis, suggesting that FAM167A has the potential for predicting BCR-ABL-independent TKI resistance, thereby guiding selection of treatment options.

Previous reports show that polymorphisms in *FAM167A* are associated with autoimmune diseases such as Sjogren’s syndrome [38, 39], systemic lupus erythematosus [40–42] and systemic sclerosis [43–45]. However, only clinical associations have been identified, and the mechanisms and pathways through which FAM167A functions in these disorders remain unknown. In addition, very little is known about FAM167A function generally. Here, we show for the first time that FAM167A induces activation of the noncanonical NF-κB pathway. Interestingly, the noncanonical NF-κB pathway regulates immunity, and its aberrant activation can promote autoimmunity [34]; thus, our findings indicate that FAM167A may cause autoimmune diseases by activating the noncanonical NF-κB pathway. In addition, this study is the first to demonstrate an association between FAM167A and cancer by identifying the role of FAM167A in TKI-resistant CML. Therefore, FAM167A may be relevant for progression or resistance of other human cancers.

We also identified DSG1 as a receptor for FAM167A. Initially, DSG1 was discovered as a component of the desmosome, which maintains tissue integrity through its function in cell adhesion [26–28]; therefore, DSG1 was presumed to be a scaffolding protein. However, further studies showed that DSG1 is not only a scaffolding protein but also a regulator of signaling pathways, including the ERK [33] and NF-κB pathways [46]; indeed, one study reported that DSG1 inhibits the NF-κB pathway [46]. Likewise, we observed that NF-κB signaling, specifically the noncanonical NF-κB pathway, is inhibited by the DSG1 complex through ubiquitination of NIK; furthermore, we demonstrated that this inhibition is blocked by FAM167A via NIK dissociation from the DSG1 complex, although the identity of the ubiquitin ligase involved in NIK ubiquitination remains unknown. Another interesting question is that of the FAM167A secretion pathway. DSG1 has a signal peptide and localizes to the cell surface through the classical pathway [47, 48], while FAM167A contains no signal peptide and is predicted to be secreted through the nonclassical pathway. Furthermore, although FAM167A was ectopically expressed in cells, FAM167A neutralizing antibody blocked the NF-κB activation, indicating that secreted FAM167A is the main inducer of NF-κB activation in CML cells. Thus, the FAM167A secretion pathway may not overlap with the pathway that mediates DSG1 surface localization. In addition, while the majority of studies on DSG1 have focused on epithelial cells [26–28, 33, 46], we validated the expression and function of DSG1 in CML cells, suggesting that DSG1 may have broader relevance in this signaling pathway in other cell types and cancers.

Previous studies show that the NF-κB pathway is critical for TKI resistance in CML [24, 25], but the detailed mechanism is unknown. Moreover, compared with the canonical NF-κB pathway, which is very well characterized, the noncanonical NF-κB pathway has received relatively limited attention [22]. Here, we show that the noncanonical NF-κB pathway is activated by FAM167A. We also discovered a role not only for the novel inducer FAM167A but also for its receptor DSG1, which regulates NIK (a central component of the noncanonical NF-κB pathway), and showed that while FAM167A activates the noncanonical NF-κB pathway, DSG1 is not just a receptor; it also inhibits this pathway. This suggests its involvement in regulating noncanonical NF-κB signaling. We also showed that targeting FAM167A is a promising strategy for treating BCR-ABL-independent TKI resistance. Our results show that FAM167A acted in both an autocrine and paracrine manner to enable CML cells to resist TKIs in vitro. However, anti-FAM167A antibody treatment reversed the TKI resistance of K562R cells. Injection of an anti-FAM167A antibody into a mouse model of CML effectively turned TKI-resistant tumors into TKI-sensitive tumors. Furthermore, while the anti-FAM167A antibody suppressed the growth of TKI-resistant tumors, there were no changes in body weight or any observable adverse effects. The functional role of FAM167A should be examined in future studies because blocking of FAM167A may have unforeseen adverse effects in vivo. Until now, no study has examined the functional role of FAM167A in vivo. Analyses of human CML samples revealed that expression of FAM167A in CD34^+^ cells from *ABL* mutation-independent imatinib-resistant patients is significantly higher than that in CD34^+^ cells from imatinib responders and *ABL* mutation-dependent imatinib-resistant patients. Based on these experimental data, anti-FAM167A antibodies may be a treatment for BCR-ABL-independent TKI-resistant patients.

## Conclusion

In this study, we provide new evidence that the FAM167A/DSG1 axis regulates the noncanonical NF-κB pathway and governs BCR-ABL-independent TKI resistance in CML. The findings show that the FAM167A-mediated noncanonical NF-κB pathway is a critical mechanism underlying BCR-ABL-independent TKI resistance in CML. Future work building on these findings could establish better approaches that offer curative treatment of CML and other diseases, including other cancers and inflammatory diseases.

## Supplementary Information

**Additional file 1: Fig. S1.** K562R cells are resistant to TKIs, while K562S cells are not. (A, B) Viability analysis of K562S and K562R cells after treatment with the indicated concentrations of imatinib (A) or nilotinib (B) for 3 days. (C, D) Flow cytometry of Annexin V-stained cells to evaluate apoptosis in K562S and K562R cells after treatment with the indicated concentrations of imatinib (C) or nilotinib (D) for 3 days. Data are representative of three (A-D) independent experiments (error bars, s.d. of triplicate (A, B) samples). Unpaired two-tailed *t*-test; ***P < 0.001. K562S, TKI-sensitive K562 cell line; K562R, BCR-ABL kinase domain mutation-free TKI-resistant K562 cell line. **Fig. S2** K562R cells exhibit BCR-ABL-independent resistance to TKIs. (A) Scheme of the sequenced region with reported mutation sites for BCR-ABL-dependent resistance. (B) Sequenced region alignment results for K562S and K562R cells. (C) Chromatograms of the reported mutation sites for BCR-ABL-dependent resistance in K562S and K562R cells. **Fig. S3** AP-1 and NF-κB are highly involved in the regulation of differentially expressed genes compared to random genes. (A, B) Proportions of genes targeted by indicated transcription factors among genes differentially expressed between K562S and K562R (A) and proportions of genes targeted by indicated transcription factors among 500 randomly selected genes (B). **Fig. S4** FAM167A increases resistance to imatinib. Viability of K562S cells transfected with the plasmid encoding the indicated gene after treatment with or without imatinib (IMA, 1 μM) for 2 days. Data are representative of two independent experiments (error bars, s.d. of triplicate samples). Unpaired two-tailed *t*-test; ***P < 0.001. **Fig. S5** FAM167A is a secreted protein. (A) In silico prediction of human, mouse, rat, and zebrafish FAM167A and HA-tagged human FAM167A secretion by the SecretomeP tool (http://www.cbs.dtu.dk/services/SecretomeP/). Proteins with an NN score above 0.5 are predicted to be secreted. (B) Immunoblot analysis of FAM167A in cell lysates and culture supernatants of K562R cells. Medium was used as a control supernatant. Supernatant proteins were concentrated by acetone precipitation. The cytoplasmic protein, Erbin, was also analyzed as a control, to confirm that culture supernatants were free from cytosolic protein. Data are representative of three (B) independent experiments. **Fig. S6** The noncanonical NF-κB pathway is activated in K562R cells. Full size image of Fig. 2H. Data are representative of three independent experiments. **Fig. S7** Identification of a FAM167A receptor. (A) Experimental design for FAM167A receptor identification. (B) Magnified images of the two distinct band regions from Fig. 3B. (C) MS/MS spectra and peptide sequence of the FAM167A band from Fig. 3B as an assay quality control. (D) Immunofluorescence microscopy analysis of DSG1 and FAM167A in K562R cell. Nuclei were visualized using Hoechst 33342. Scale bars, 10 μm. (E) NF-κB luciferase reporter activity in K562S cells 24 h after transfection of the plasmid encoding FAM167A and treatment with indicated concentrations of anti-FAM167A neutralizing antibody. Data are representative of two (D, E) independent experiments (error bars, s.d. of triplicate (E) samples). Unpaired two-tailed *t*-test; *P < 0.05. **Fig. S8** FAM167A regulates the noncanonical NF-κB pathway through DSG1. (A) Surface DSG1 staining of K562R cells after DSG1 knockdown using recombinant lentiviruses encoding two different DSG1-specific shRNAs with an anti-DSG1 antibody. (B) qRT-PCR analysis of Erbin mRNA in K562S, K562R, and K562R cells treated with 1 μM imatinib for 24 h. (C) Immunoblot analysis for Erbin in K562S and K562R cells. Data are representative of three (A-C) independent experiments (error bars, s.d. of triplicate (B) samples). Unpaired two-tailed *t*-test; NS, not significant. **Fig. S9** The candidate ubiquitin ligase components TRAF3, CHIP, and c-cbl do not bind to DSG1. (A) Coimmunoprecipitation analysis of DSG1, Erbin, NIK, TRAF3, CHIP, and c-cbl in K562S and K562R cells. (B) Coimmunoprecipitation analysis of DSG1, NIK, and TRAF3 in K562S cells using anti-DSG1 and anti-TRAF3 antibodies. (C) Coimmunoprecipitation analysis of DSG1, NIK, and CHIP in K562S cells using anti-DSG1 and anti-CHIP antibodies. (D) Coimmunoprecipitation analysis of DSG1, NIK, and c-cbl in K562S cells using anti-DSG1 and anti-c-cbl antibodies. GAPDH was used as an internal standard (A). Data are representative of three (A-D) independent experiments. **Fig. S10** FAM167A levels are elevated in KCL22R cells with BCR-ABL-independent TKI resistance. (A) Scheme of the sequenced region with reported mutation sites for BCR-ABL-dependent resistance. (B) Sequenced region alignment results for KCL22S and KCL22R cells. (C) Chromatograms of the reported mutation sites for BCR-ABL-dependent resistance in KCL22S and KCL22R cells. (D) qRT-PCR analysis of FAM167A mRNA in KCL22S, KCL22R, and KCL22R cells treated with 1 μM imatinib for 24 h. Data are representative of three (D) independent experiments (error bars, s.d. of triplicate (D) samples). Unpaired two-tailed *t*-test; **P < 0.01, ***P < 0.001. **Table S1.** CML patient characteristics for the samples used in this study.

## Data Availability

All data used and/or performed in this study are available from the corresponding author upon reasonable request.

## Abbreviations

TKI: Tyrosine kinase inhibitor
CML: Chronic myeloid leukemia
DSG1: Desmoglein-1
NF-κB: Nuclear factor-κB
NIK: NF-κB-inducing kinase
qRT-PCR: Quantitative reverse transcription PCR
SDS: Sodium dodecyl sulfate
EMSA: Electrophoretic mobility shift assay
HE: Hematoxylin and eosin
LPS: Lipopolysaccharide
NIK-DN: Dominant-negative NIK
shRNA: Short hairpin RNA
