# Supplementary material for: FAM167A is a key molecule to induce BCR-ABL-independent TKI resistance in CML via noncanonical NF-κB signaling activation
Source: J Exp Clin Cancer Res. 2022 Mar 3;41:82. doi: 10.1186/s13046-022-02298-1 (PMC8892744; doi:10.1186/s13046-022-02298-1)
Supplement: Supplementary file 1 — Additional file 1: Fig. S1. K562R cells are resistant to TKIs, while K562S cells are not. (A, B) Viability analysis of K562S and K562R cells after treatment with the indicated concentrations of imatinib (A) or nilotinib (B) for 3 days. (C, D) Flow cytometry of Annexin V-stained cells to evaluate apoptosis in K562S and K562R cells after treatment with the indicated concentrations of imatinib (C) or nilotinib (D) for 3 days. Data are representative of three (A-D) independent experiments (error bars, s.d. of triplicate (A, B) samples). Unpaired two-tailed t-test; ***P < 0.001. K562S, TKI-sensitive K562 cell line; K562R, BCR-ABL kinase domain mutation-free TKI-resistant K562 cell line. Fig. S2 K562R cells exhibit BCR-ABL-independent resistance to TKIs. (A) Scheme of the sequenced region with reported mutation sites for BCR-ABL-dependent resistance. (B) Sequenced region alignment results for K562S and K562R cells. (C) Chromatograms of the reported mutation sites for BCR-ABL-dependent resistance in K562S and K562R cells. Fig. S3 AP-1 and NF-κB are highly involved in the regulation of differentially expressed genes compared to random genes. (A, B) Proportions of genes targeted by indicated transcription factors among genes differentially expressed between K562S and K562R (A) and proportions of genes targeted by indicated transcription factors among 500 randomly selected genes (B). Fig. S4 FAM167A increases resistance to imatinib. Viability of K562S cells transfected with the plasmid encoding the indicated gene after treatment with or without imatinib (IMA, 1 μM) for 2 days. Data are representative of two independent experiments (error bars, s.d. of triplicate samples). Unpaired two-tailed t-test; ***P < 0.001. Fig. S5 FAM167A is a secreted protein. (A) In silico prediction of human, mouse, rat, and zebrafish FAM167A and HA-tagged human FAM167A secretion by the SecretomeP tool (http://www.cbs.dtu.dk/services/SecretomeP/). Proteins with an NN score above 0.5 are predicted to [file 13046_2022_2298_MOESM1_ESM.docx]

**Additional file 1**

**FAM167A is a key molecule to induce BCR-ABL-independent TKI resistance in CML via noncanonical NF-κB signaling activation**

Taewoo Yang^1,2^, Kyu-Young Sim^1,2^, Gwang-Hoon Ko^1,2^, Jae-Sook Ahn^3^, Hyeoung-Joon Kim^3^, and Sung-Gyoo Park^1^

^1^Institute of Pharmaceutical Sciences, College of Pharmacy, Seoul National University, Seoul 08826, Republic of Korea

^2^School of Life Sciences, Gwangju Institute of Science and Technology, Gwangju 61005, Republic of Korea

^3^Department of Hematology-Oncology, Chonnam National University Hwasun Hospital, Hwasun 58128, Republic of Korea

^*^Correspondence and requests for materials should be addressed to S.G.P. (email: riceo2@snu.ac.kr)


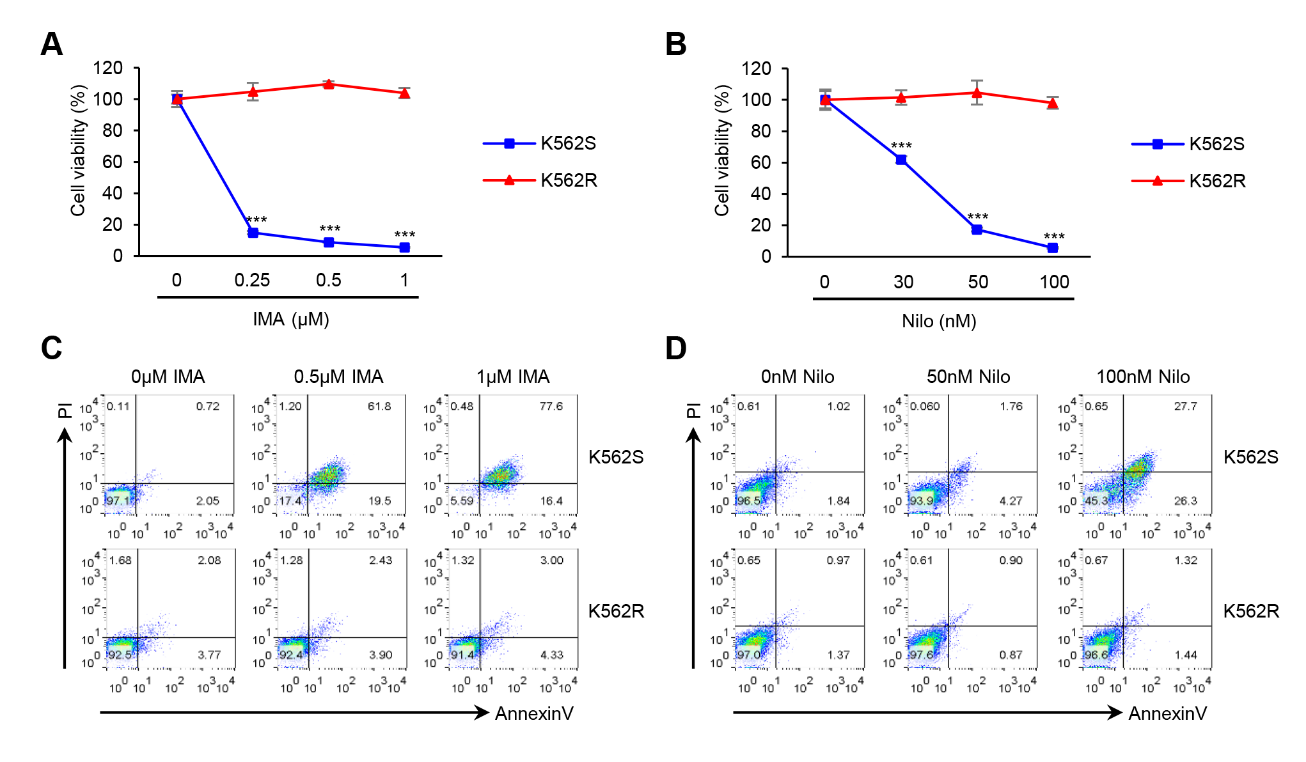


**Fig. S1 K562R cells are resistant to TKIs, while K562S cells are not.**

**A**, **B** Viability analysis of K562S and K562R cells after treatment with the indicated concentrations of imatinib (**A**) or nilotinib (**B**) for 3 days. **C**, **D** Flow cytometry of Annexin V-stained cells to evaluate apoptosis in K562S and K562R cells after treatment with the indicated concentrations of imatinib (**C**) or nilotinib (**D**) for 3 days. Data are representative of three (**A**−**D**) independent experiments (error bars, s.d. of triplicate (**A**, **B**) samples). Unpaired two-tailed *t*-test; ****P*<0.001. K562S, TKI-sensitive K562 cell line; K562R, BCR-ABL kinase domain mutation-free TKI-resistant K562 cell line.

**
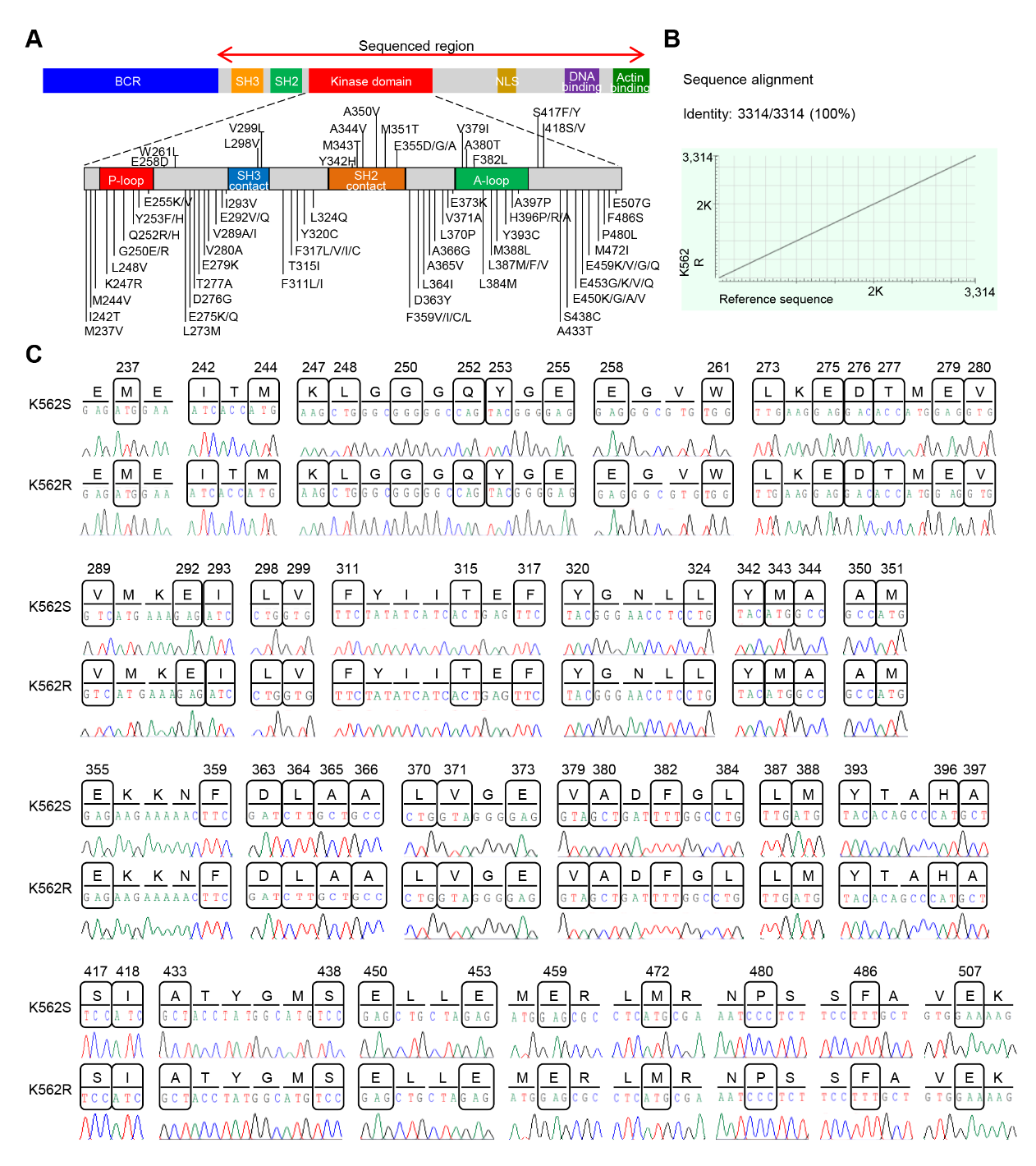
**

**Fig. S2 K562R cells exhibit BCR-ABL-independent resistance to TKIs.**

**A** Scheme of the sequenced region with reported mutation sites for BCR-ABL-dependent resistance. **B** Sequenced region alignment results for K562S and K562R cells. **C** Chromatograms of the reported mutation sites for BCR-ABL-dependent resistance in K562S and K562R cells.

**
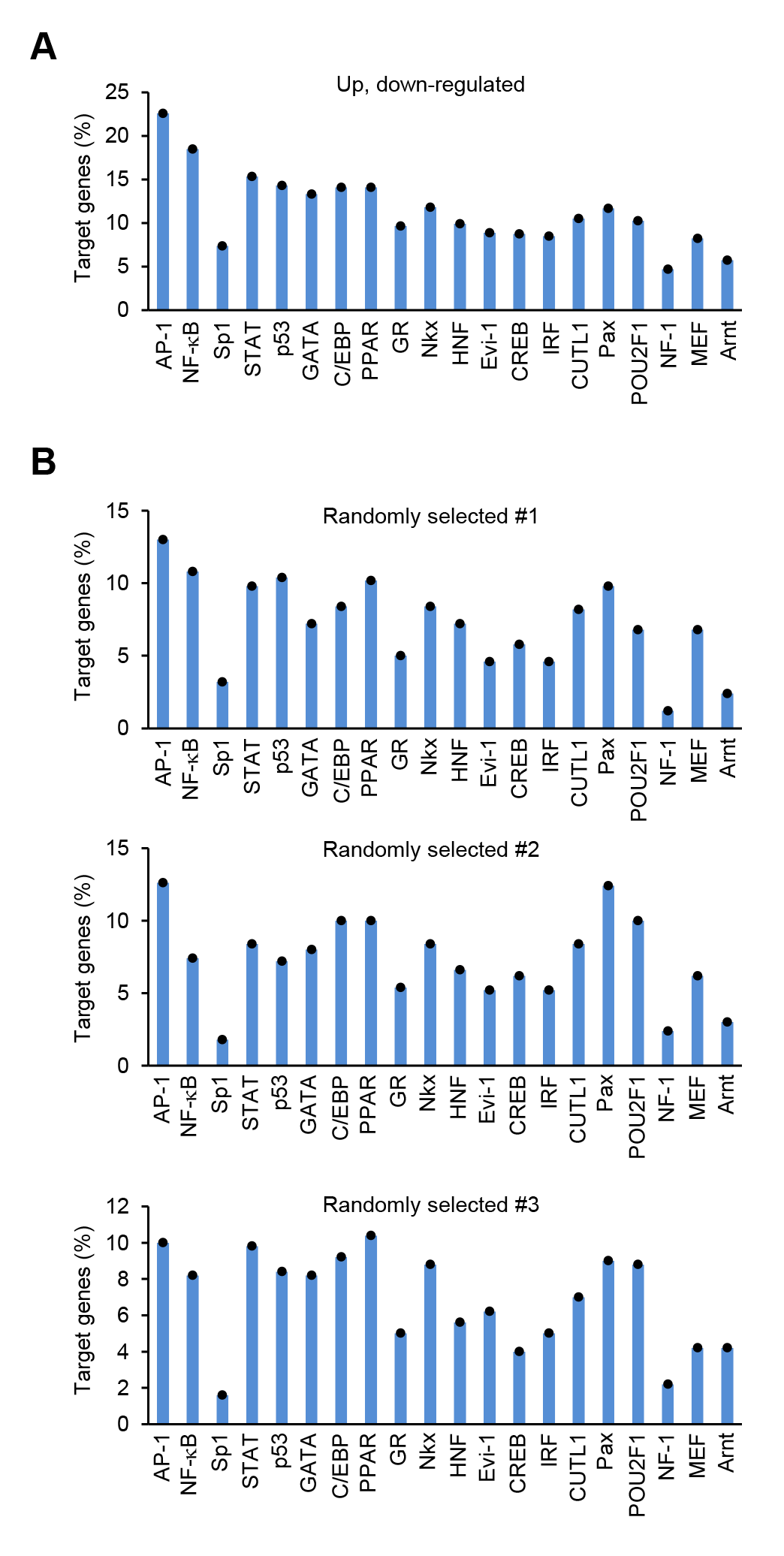
**

**Fig. S3 AP-1 and NF-κB are highly involved in the regulation of differentially expressed genes compared to random genes.**

**A**, **B** Proportions of genes targeted by indicated transcription factors among genes differentially expressed between K562S and K562R (**A**) and proportions of genes targeted by indicated transcription factors among 500 randomly selected genes (**B**).

**
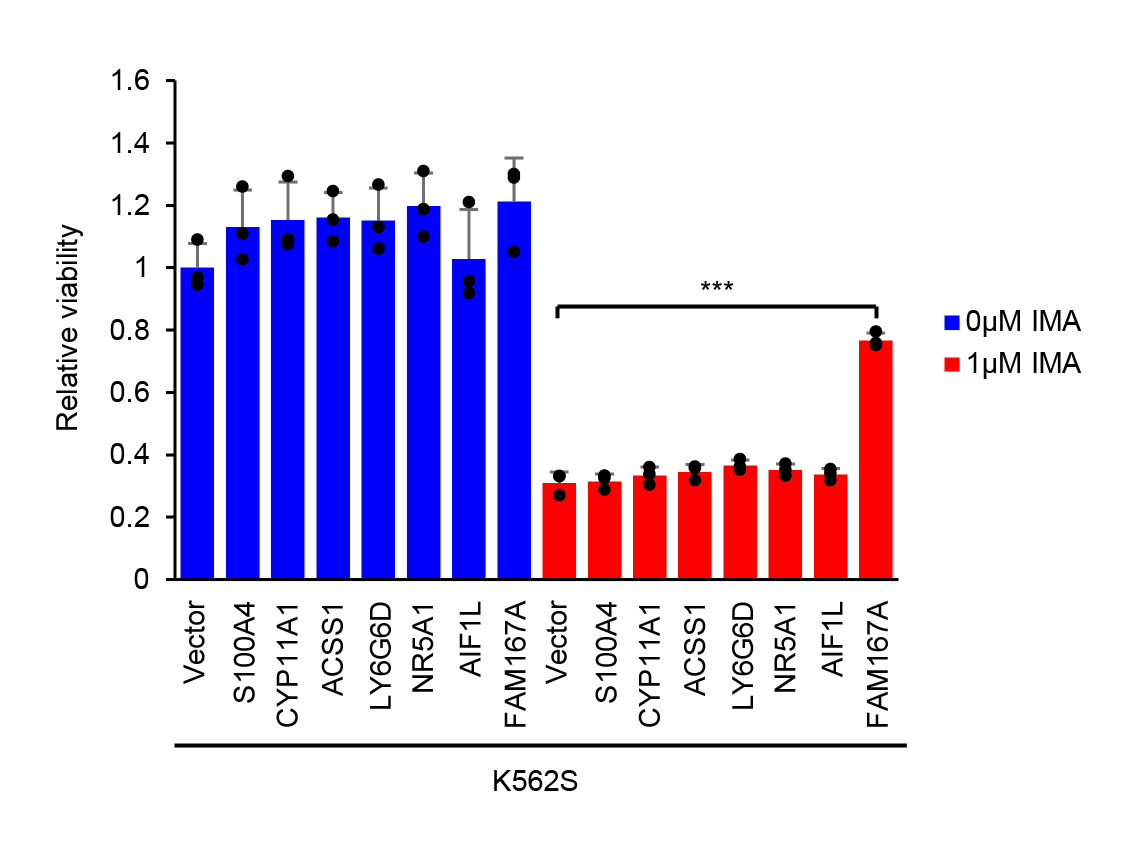
**

**Fig. S4 FAM167A increases resistance to imatinib.**

Viability of K562S cells transfected with the plasmid encoding the indicated gene after treatment with or without imatinib (IMA, 1 µM) for 2 days. Data are representative of two independent experiments (error bars, s.d. of triplicate samples). Unpaired two-tailed *t*-test; ****P*<0.001.

**
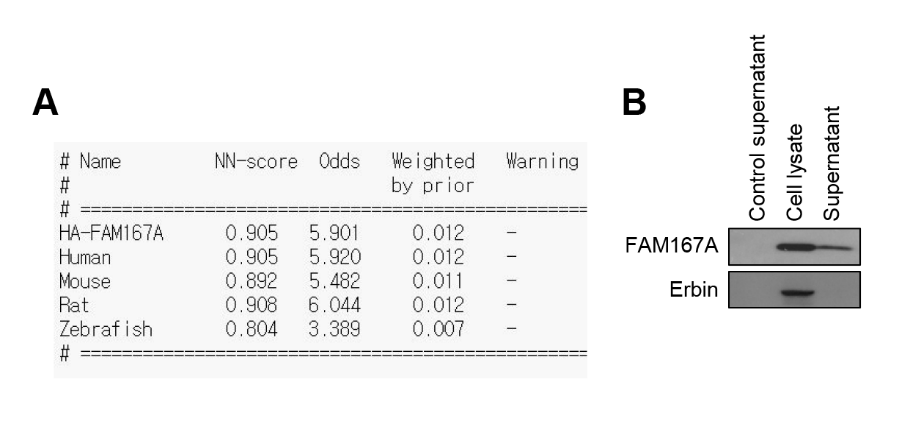
**

**Fig. S5 FAM167A is a secreted protein.**

**A** *In silico* prediction of human, mouse, rat, and zebrafish FAM167A and HA-tagged human FAM167A secretion by the SecretomeP tool (http://www.cbs.dtu.dk/services/SecretomeP/). Proteins with an NN score above 0.5 are predicted to be secreted. **B** Immunoblot analysis of FAM167A in cell lysates and culture supernatants of K562R cells. Medium was used as a control supernatant. Supernatant proteins were concentrated by acetone precipitation. The cytoplasmic protein, Erbin, was also analyzed as a control, to confirm that culture supernatants were free from cytosolic protein. Data are representative of three (**B**) independent experiments.

**
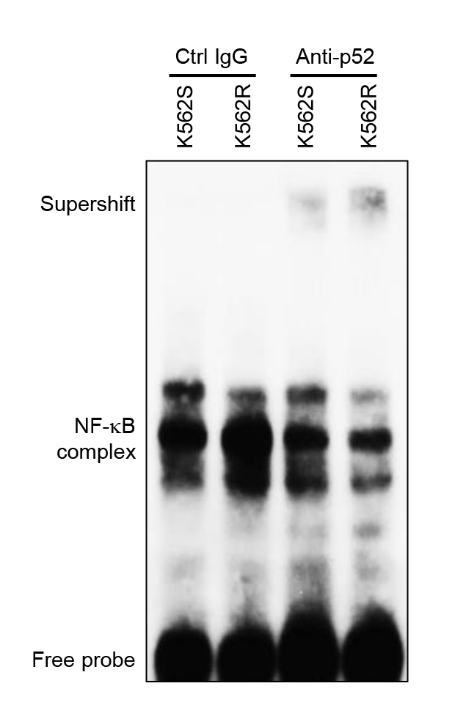
**

**Fig. S6 The noncanonical NF-κB pathway is activated in K562R cells.**

Full size image of **Fig. 2H**. Data are representative of three independent experiments.

**
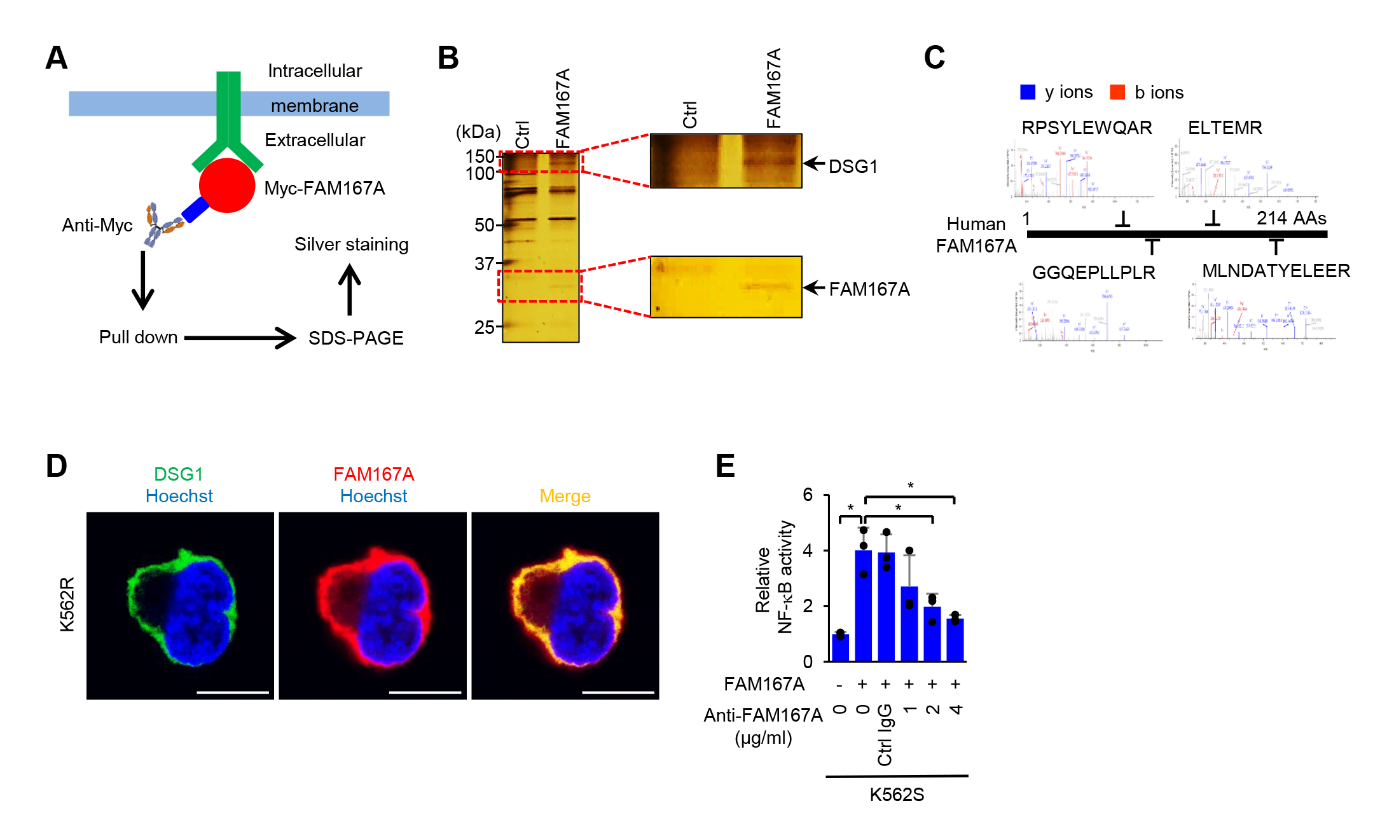
**

**Fig. S7 Identification of a FAM167A receptor.**

**A** Experimental design for FAM167A receptor identification. **B** Magnified images of the two distinct band regions from **Fig. 3B**. **C** MS/MS spectra and peptide sequence of the FAM167A band from **Fig. 3B** as an assay quality control. **D** Immunofluorescence microscopy analysis of DSG1 and FAM167A in K562R cell. Nuclei were visualized using Hoechst 33342. Scale bars, 10 µm. **E** NF-κB luciferase reporter activity in K562S cells 24 h after transfection of the plasmid encoding FAM167A and treatment with indicated concentrations of anti-FAM167A neutralizing antibody. Data are representative of two (**D**, **E**) independent experiments (error bars, s.d. of triplicate (**E**) samples). Unpaired two-tailed *t*-test; **P*<0.05.

**
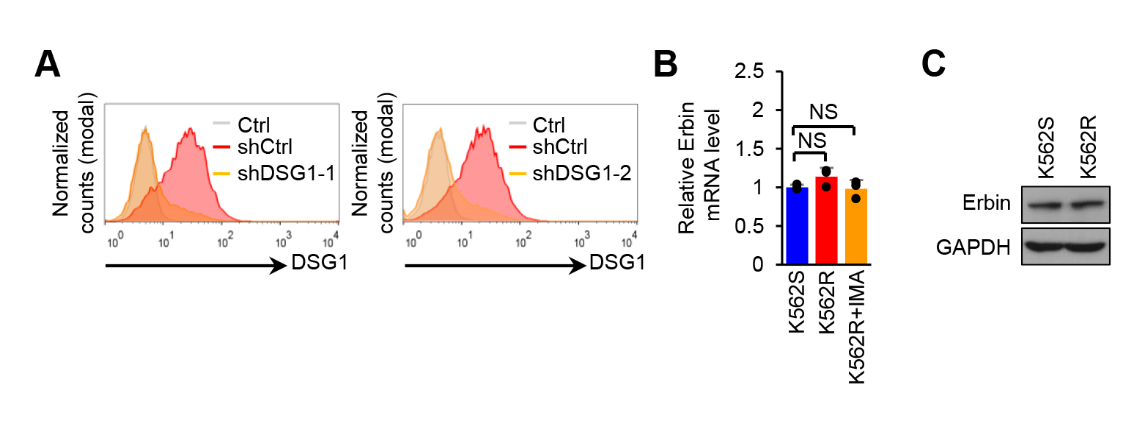
**

**Fig. S8 FAM167A regulates the noncanonical NF-κB pathway through DSG1.**

**A** Surface DSG1 staining of K562R cells after DSG1 knockdown using recombinant lentiviruses encoding two different DSG1-specific shRNAs with an anti-DSG1 antibody. **B** qRT-PCR analysis of Erbin mRNA in K562S, K562R, and K562R cells treated with 1 µM imatinib for 24 h. **C** Immunoblot analysis for Erbin in K562S and K562R cells. Data are representative of three (**A**−**C**) independent experiments (error bars, s.d. of triplicate (**B**) samples). Unpaired two-tailed *t*-test; NS, not significant.

**
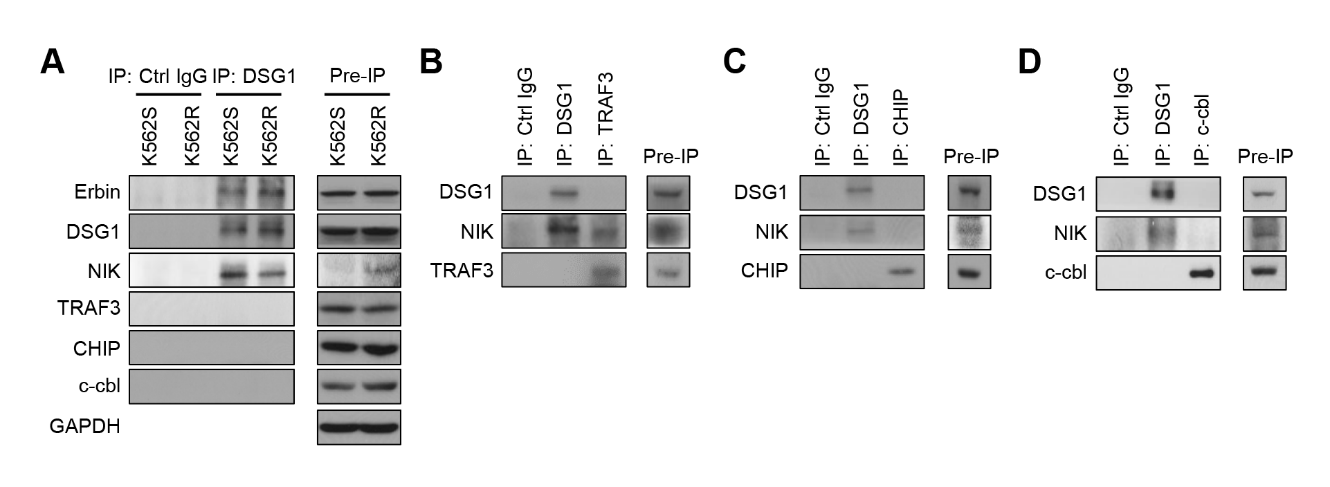
**

**Fig. S9 The candidate ubiquitin ligase components TRAF3, CHIP, and c-cbl do not bind to DSG1.**

**A** Coimmunoprecipitation analysis of DSG1, Erbin, NIK, TRAF3, CHIP, and c-cbl in K562S and K562R cells. **B** Coimmunoprecipitation analysis of DSG1, NIK, and TRAF3 in K562S cells using anti-DSG1 and anti-TRAF3 antibodies. **C** Coimmunoprecipitation analysis of DSG1, NIK, and CHIP in K562S cells using anti-DSG1 and anti-CHIP antibodies. **D** Coimmunoprecipitation analysis of DSG1, NIK, and c-cbl in K562S cells using anti-DSG1 and anti-c-cbl antibodies. GAPDH was used as an internal standard (**A**). Data are representative of three (**A**−**D**) independent experiments.

**
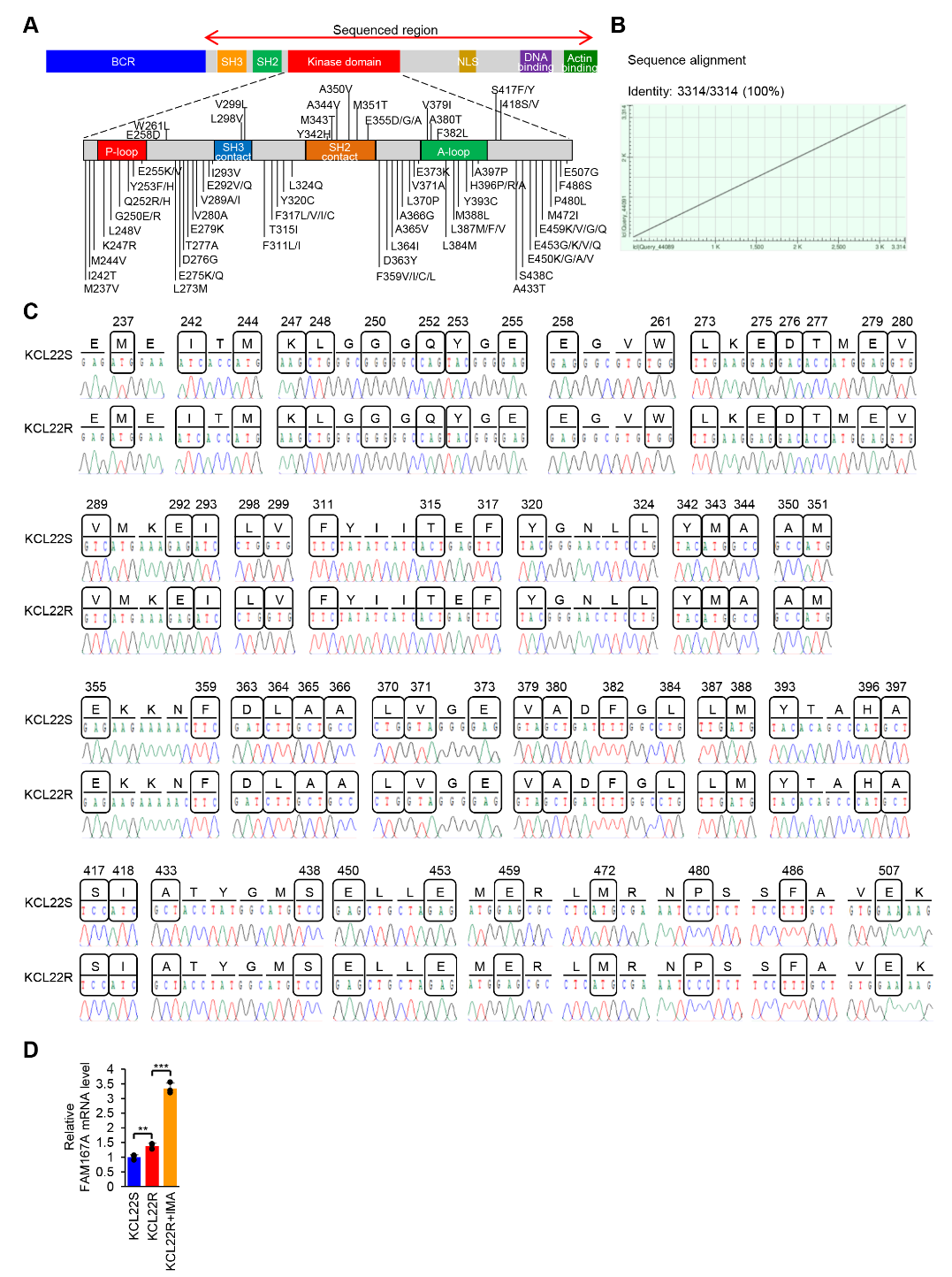
**

**Fig. S10 FAM167A levels are elevated in KCL22R cells with BCR-ABL-independent TKI resistance.**

**A** Scheme of the sequenced region with reported mutation sites for BCR-ABL-dependent resistance. **B** Sequenced region alignment results for KCL22S and KCL22R cells. **C** Chromatograms of the reported mutation sites for BCR-ABL-dependent resistance in KCL22S and KCL22R cells. **D** qRT-PCR analysis of FAM167A mRNA in KCL22S, KCL22R, and KCL22R cells treated with 1 µM imatinib for 24 h. Data are representative of three (**D**) independent experiments (error bars, s.d. of triplicate (**D**) samples). Unpaired two-tailed *t*-test; ***P*<0.01, ****P*<0.001.

**Table S1. CML patient characteristics for the samples used in this study.**

| **Patient No.** | **Age** | **Sex** | **Dignosis** | **Imatinib resistance^1^** | **BCR-ABL mutation status^2^** |
| --- | --- | --- | --- | --- | --- |
| **Imatinib responsive patients** | | | | | |
| 1 | 74 | F | CP | Responsive | - |
| 2 | 59 | F | CP | Responsive | - |
| 3 | 68 | F | CP | Responsive | - |
| 4 | 67 | F | CP | Responsive | - |
| 5 | 69 | M | CP | Responsive | - |
| 6 | 60 | M | CP | Responsive | - |
| 7 | 57 | M | CP | Responsive | - |
| 8 | 48 | M | CP | Responsive | - |
| 9 | 48 | M | CP | Responsive | - |
| 10 | 41 | M | CP | Responsive | - |
| 11 | 56 | M | CP | Responsive | - |
| 12 | 75 | F | CP | Responsive | - |
| 13 | 56 | M | CP | Responsive | - |
| 14 | 46 | M | CP | Responsive | - |
| 15 | 69 | M | CP | Responsive | - |
| 16 | 76 | F | CP | Responsive | - |
| 17 | 69 | F | CP | Responsive | - |
| 18 | 23 | M | CP | Responsive | - |
| 19 | 69 | F | CP | Responsive | - |
| 20 | 53 | F | CP | Responsive | - |
| 21 | 67 | M | CP | Responsive | - |
| 22 | 61 | M | CP | Responsive | - |
| 23 | 70 | M | CP | Responsive | - |
| 24 | 73 | F | CP | Responsive | - |
| 25 | 26 | M | CP | Responsive | - |
| 26 | 18 | M | CP | Responsive | - |
| 27 | 52 | M | CP | Responsive | - |
| 28 | 58 | M | CP | Responsive | - |
| 29 | 66 | M | CP | Responsive | - |
| 30 | 34 | M | CP | Responsive | - |
| 31 | 79 | F | CP | Responsive | - |
| 32 | 53 | M | CP | Responsive | - |
| 33 | 43 | M | CP | Responsive | - |
| 34 | 43 | F | CP | Responsive | - |
| **Imatinib resistant patients lacking BCR-ABL mutation** | | | | | |
| 1 | 62 | M | CP | Resistant | No mutation |
| 2 | 37 | F | CP | Resistant | No mutation |
| 3 | 44 | M | CP | Resistant | No mutation |
| 4 | 77 | M | CP | Resistant | No mutation |
| 5 | 61 | F | CP | Resistant | No mutation |
| 6 | 37 | F | CP | Resistant | No mutation |
| 7 | 56 | M | CP | Resistant | No mutation |
| 8 | 28 | M | CP | Resistant | No mutation |
| 9 | 49 | M | CP | Resistant | No mutation |
| 10 | 20 | F | AP | Resistant | No mutation |
| 11 | 53 | F | CP | Resistant | No mutation |
| 12 | 52 | M | AP | Resistant | No mutation |
| **Imatinib resistant patients harboring BCR-ABL mutation** | | | | | |
| 1 | 51 | M | CP | Resistant | Y253H |
| 2 | 61 | F | CP | Resistant | G250E |
| 3 | 60 | F | AP | Resistant | F359I |
| 4 | 40 | M | BP | Resistant | T315I |
| 5 | 43 | F | CP | Resistant | P480L |
| 6 | 44 | M | CP | Resistant | H396R |
| 7 | 58 | M | CP | Resistant | Y215F |
| 8 | 30 | M | CP | Resistant | F317L |
| 9 | 34 | M | CP | Resistant | E255K |
| 10 | 61 | M | BP | Resistant | E255K |
| 11 | 60 | F | CP | Resistant | T315I |
| 12 | 54 | M | CP | Resistant | G250E |

^1^Imatinib resistance were classified clinically based on the European Leukemia Net guidelines. ^2^Mutational status of BCR-ABL were confirmed by sequencing analysis

CP, chronic phase; AP, accelerated phase; BP, blastic phase
